# Around the World in Eight Million Years: Historical Biogeography and Evolution of the Spray Zone Spider *Amaurobioides* (Araneae: Anyphaenidae)

**DOI:** 10.1371/journal.pone.0163740

**Published:** 2016-10-12

**Authors:** F. Sara Ceccarelli, Brent D. Opell, Charles R. Haddad, Robert J. Raven, Eduardo M. Soto, Martín J. Ramírez

**Affiliations:** 1 División de Aracnología, Museo Argentino de Ciencias Naturales, Av. Angel Gallardo 470, C1405DJR, Buenos Aires, Argentina; 2 Department of Biological Sciences, 1405 Perry Street, Virginia Tech, Blacksburg, VA 24061, United States of America; 3 Dept. of Zoology & Entomology, University of the Free State, P. O. Box 339, Bloemfontein 9300, South Africa; 4 Arachnid Collection, Terrestrial Biodiversity Group, Queensland Museum, Grey St, P. O. Box 3300, South Brisbane 4101, Queensland, Australia; 5 Departamento de Ecología, Genética y Evolución, IEGEBA (CONICET-UBA), Facultad de Ciencias Exactas y Naturales, Universidad de Buenos Aires, Ciudad Universitaria, Pabellón II (C1428 EHA), Buenos Aires, Argentina; Scientific Research Centre of the Slovenian Academy of Sciences and Art, SLOVENIA

## Abstract

Closely related organisms with transoceanic distributions have long been the focus of historical biogeography, prompting the question of whether long-distance dispersal, or tectonic-driven vicariance shaped their current distribution. Regarding the Southern Hemisphere continents, this question deals with the break-up of the Gondwanan landmass, which has also affected global wind and oceanic current patterns since the Miocene. With the advent of phylogenetic node age estimation and parametric bioinformatic advances, researchers have been able to disentangle historical evolutionary processes of taxa with greater accuracy. In this study, we used the coastal spider genus *Amaurobioides* to investigate the historical biogeographical and evolutionary processes that shaped the modern-day distribution of species of this exceptional genus of spiders. As the only genus of the subfamily Amaurobioidinae found on three Southern Hemisphere continents, its distribution is well-suited to study in the context of Gondwanic vicariance versus long-distance, transoceanic dispersal. Ancestral species of the genus *Amaurobioides* appear to have undergone several long-distance dispersal events followed by successful establishments and speciation, starting from the mid-Miocene through to the Pleistocene. The most recent common ancestor of all present-day *Amaurobioides* species is estimated to have originated in Africa after arriving from South America during the Miocene. From Africa the subsequent dispersals are likely to have taken place predominantly in an eastward direction. The long-distance dispersal events by *Amaurobioides* mostly involved transoceanic crossings, which we propose occurred by rafting, aided by the Antarctic Circumpolar Current and the West Wind Drift.

## Introduction

One of the main intrigues in biogeography has always been explaining the distribution and evolution of closely related groups of terrestrial organisms on landmasses separated by vast expanses of ocean, such as oceanic islands or separate continents. In the case of islands that formed from the seafloor, their current biota represents local colonisations and in some cases radiations. Studies on the biota of remote oceanic islands, especially of volcanic origin, have been exemplary in demonstrating the ability of different organisms in colonising new areas following transoceanic long-distance dispersal events [1–3]. On the other hand, landmasses that were separated by tectonism may harbour biota that is the result of vicariant speciation, or species may have moved between the landmasses by over-water/aerial dispersal and evolved *in situ*. An example of such landmasses are the fragments of the supercontinent Gondwana, which began breaking up during the early Jurassic, approximately 190 million years ago [4]. The separation of the last part of the Gondwanan landmass, namely Australia-Antarctica-South America during the Miocene, gave rise to the Antarctic Circumpolar Current in the ocean and the West Wind Drift in the atmosphere [5]. The Antarctic Circumpolar Current and West Wind Drift have been key factors affecting the directionality of long-distance dispersal events in the Southern Hemisphere, particularly for organisms that disperse by rafting (or oceanic drift) and wind, creating what has been termed “dispersal asymmetry” for a predominantly eastward dispersal pattern [6–9].

Spiders, amongst the arachnids, are generally considered of high vagility due to their dispersal capacity by the phenomenon termed ballooning, by which juveniles can be carried long distances through the air by a strand of silk released from their spinnerets (see [10]). Not only can many spiders undergo long-distance dispersal by this mechanism, but some species have also been shown to withstand contact with water, and even take-off from the surface of salty, turbulent water [11], a crucial feature for surviving transoceanic dispersal. Studies of spiders on different oceanic islands have shown that they are among the first and most successful colonisers, with the lineages at times arising from multiple dispersal events [12–15] (Soto pers. obs.). However, for some spiders and other arachnids, which lack the propensity for ballooning, an alternative explanation must be provided for groups with a transoceanic (or volcanic island) distribution. Certain globally distributed groups of arachnids with poor dispersal capacities (e.g. opilionids, [16], or palpimanoid spiders, [17]) have diverged through tectonic related vicariant cladogenesis, yet in other cases the divergence times do not coincide with landmass fragmentations [18]. As the alternative to aerial dispersal, rafting must also be considered for such spiders. Spiders have been observed on vegetation (macrophyte) rafts in the Amazon [19] and rafting as a means of transoceanic dispersal has been suggested for mygalomorphs [20], trapdoor spiders [21], salticids [22], the genus *Dysdera* [23], the desid *Desis marina* [24] and the anyphaenid genus *Amaurobioides* O. Pickard-Cambridge, 1883 [25, 26].

Among the spiders, several species of the family Anyphaenidae are known to disperse through ballooning in the Northern Hemisphere [27, 28], but not *Amaurobioides* (Ramírez, pers. obs.; see below). The anyphaenid genus *Amaurobioides* is exceptional for its ecology and distribution. According to the detailed study by Lamoral [29], *Amaurobioides africana* Hewitt, 1917 seal their retreats to endure daily periods of immersion as the tides rise. With low tide they open the entrance of their silken cells, and at night prey mostly on isopod and amphipod crustaceans. The individuals prey from the cell entrance, not walking away from their retreat as other spiders do. The species of *Amaurobioides* from Chile, Australia and New Zealand position their retreats in the spray- rather than the intertidal zone, thus are probably immersed less often than the population studied by Lamoral, but still seal their retreats in a similar way [30–33]. To date, 12 species have been described from rocky shores in South Africa (one species), Australia (two species), New Zealand (eight species) and Chile (one species) [29, 30]. However, the number of species from New Zealand is probably over-estimated, since there appear to be at most three genetic lineages [32, 33].

While reviewing the spiders of the subantarctic islands of New Zealand, Forster [24] thought that *Amaurobioides* was composed of a single species widespread in South Africa, Australia and New Zealand, that dispersed easily across sea expanses. He later revised the genus, finding morphological differences to distinguish no fewer than 10 species, thus suggesting that their dispersal ability has been overestimated [30]. Later on, Forster & Forster [34] attributed their Gondwanic distribution to ancient vicariance, while Hewitt [35] had mentioned the possibility of the species’ passive dispersal on floating seaweed. While the distribution of this genus is Gondwanan in the sense that they are found on continental shelves once forming part of Gondwana, the question that arises is whether this distributional pattern can be attributed to ancient vicariance from the separation of the landmasses or whether more recent, long-distance dispersal events were involved in the species’ distributions. On a smaller scale, phylogeographic studies of *Amaurobioides* from Australia and New Zealand have revealed their propensity for dispersal between the two landmasses [26], mainly from Australia towards New Zealand.

Here, we present a parametric biogeographical study of *Amaurobioides* species from Africa, Australia, New Zealand and South America based on molecular phylogenetic species tree analyses performed using two mitochondrial (cytochrome oxidase c subunit 1 and 16S rDNA) and two nuclear (Histone 3-a and 28S r-DNA) gene fragments, to shed light on the genus’ geographic range evolution and compare it to other global biogeographical and evolutionary processes.

## Materials and Methods

### Taxon Sampling

A total of 45 *Amaurobioides* individuals were used for molecular work in this study, of which 34 were assigned to the species *A*. *africana* (20 individuals from South Africa), *A*. *chilensis* (3 individuals from Chile), *A*. *isolata* Hirst, 1993 (1 individual from South Australia), *A*. *litoralis* Hickman, 1949 (1 individual from Tasmania, Australia), *A*. *maritima* (2 individuals from New Zealand), *A*. *pallida* Forster, 1970 (3 individuals from New Zealand) and *A*. *pleta* Forster, 1970 (4 individuals from New Zealand). The remaining 11 individuals belonged to undescribed species from South Africa (7 individuals) and Flinders Island, Tasmania (4 individuals). This sampling covers almost the entire known distributional range of the genus, with the exception of the subantarctic Auckland and Campbell islands of New Zealand, where *A*. *piscator* Hogg, 1909 is found. Collecting details of all *Amaurobioides* individuals used for molecular work can be found in Table A in S1 File. Collecting and export permits for New Zealand were issued by New Zealand’s Department of Conservation. For *Amaurobioides* species collected in Australia, the South Australian Department of Environment and Natural Resources and the Tasmanian Department of Primary Industries, Parks, Water, and Environment issued collecting permits, and the Australian Wildlife Trade Assessments provided the export permit. Further specimens from Tasmania were collected on Bush Blitz (www.bushblitz.org.au) expeditions with National Park permits provided by Tasmanian National Parks and Wildlife Service. For the South African specimens, collecting permits were obtained from CapeNature (Western Cape Province) and the Eastern Cape Department of Economic Development and Environmental Affairs (Eastern Cape Province). *Amaurobioides chilensis* was collected in unprotected public areas in Chile, where permits are not needed. None of the field studies involved endangered or protected species.

As sister- and outgroup taxa for *Amaurobioides*, we selected 60 anyphaenid taxa based on previous molecular phylogenetic studies (Soto pers. obs.) [36]. We chose one specimen per species of the following anyphaenid genera belonging to the tribe Amaurobioidini of the subfamily Amaurobioidinae: *Acanthoceto* (5 species), *Axyracrus* (1 species, monotypic), *Aysenia* (3 species), *Aysenoides* (4 species), *Coptoprepes* (3 species), *Ferrieria* (1 species, monotypic), *Gamakia* (1 species, monotypic), *Negayan* (3 species), and *Selknamia* (1 species, monotypic). From the subfamily Amaurobioidinae, tribe Gayennini, we chose one specimen per genus for the genera *Arachosia*, *Araiya*, *Gayenna*, *Gayennoides*, *Monapia*, *Oxysoma*, *Phidyle*, *Tasata* and *Tomopisthes*, two species belonging to *Sanogasta* and one specimen per species from the genus *Philisca* (15 species), to ensure that the age of the mrca of *Philisca* species endemic to Juan Fernandez Island was less than that of the island (Soto pers. obs.). The genus *Josa*, a member of Amaurobioidinae not assigned to a tribe, was represented by *J*. *calilegua*, *J*. *riveti* and an undescribed species (*Josa* sp.). Additionally, 11 anyphaenid species belonging to nine genera from the subfamily Anyphaeninae were used as outgroup taxa and for fossil node age constraints (see below): *Anyphaena*, *Anyphaenoides*, *Aysha*, *Buckupiella*, *Hatitia*, *Hibana*, *Jessica*, *Otoniela* and *Xiruana*. A specimen belonging to the clubionid genus *Elaver* was used for rooting the trees, following the molecular results of the Spider Tree of Life project (Ramírez *et al*., in prep), which show that the Chilean genus *Malenella*, formerly considered a member of Anyphaenidae, belongs elsewhere. For molecular phylogenetic analyses, a combination of DNA sequences from previous studies (Soto pers. obs.) [36] and sequences generated *de novo* was used (for details of specimens and sequences used please see Table A in S1 File).

### DNA extraction, PCR and Sequencing

For the sequences generated *de novo* for this study, DNA was extracted from leg muscle tissue using the Qiagen DNeasy Blood and Tissue Kit, following the manufacturer’s protocol and digesting the tissue at 56°C over-night with Proteinase K. Polymerase Chain Reaction (PCR) mixes contained 1.5μl x10 PCR Buffer (Thermo Scientific), 10 μmoles MgCl_2_, 0.25 μmoles of each dNTP, 0.4 μmoles of each primer, 0.1 μl Taq Polymerase (Thermo Scientific), 0.5 μl BSA, 1–2 μl genomic DNA and ddH_2_O to bring the final volume to 15 μl. The primers used for amplification can be found in Table B in S1 File. Thermal cycling profiles included an initial denaturing step at 95°C for 3 minutes, followed by 15 cycles of 30 seconds at 95°C, 30 seconds at the annealing temperature (51°C for nuclear and 45°C for mitochondrial gene fragments) and 45 minutes at 72°C; an additional 20 cycles were then run with the annealing temperature lowered by 3°C. A final extension step of 10 minutes at 72°C was then set. PCR products were purified using ExosAP (Thermo Scientific) following the manufacturer’s indications and sent for sequencing to Macrogen Inc., Korea. Sequences were edited based on the chromatograms in Sequencher v. 4.1.4 (GeneCodes corp.), where each protein-coding gene fragment was checked for stop-codons (indicating possible pseudogenes).

### Phylogenetic Inference and Divergence Dating

The edited sequences and the sequences obtained from previous studies were aligned to produce one data matrix per gene fragment. Matrices for divergence dating included all of the outgroups mentioned previously to allow for node age calibrations, whereas the remaining analyses were carried out with *Aysenia elongata* Tullgren, 1902 and *Coptoprepes campanensis* Ramírez, 2003 as the only outgoups. Alignment for the Histone 3-a (henceforth H3a) gene fragment was straightforward and was carried out in the online version of MAFFT v. 7 [37] using the “Auto” strategy and a gap opening penalty of 1.53. The cytochrome oxidase c subunit 1 (henceforth COI) gene fragment contained indels and was therefore aligned based on its translation to protein in TranslatorX [38] while for the 16S rDNA (henceforth 16S) and 28S r-DNA (henceforth 28S) gene fragments the online version of T-coffee was used [39, 40] using the default settings, that take into account secondary structure of ribosomal DNA during alignment. Poorly aligned positions for the 16S matrix were removed using the Gblocks server [41] allowing for gap positions and less strict flanking positions in the final alignment. Furthermore, recombination in nuclear genes has been shown to interfere with reliable topological inferences [42], so we tested interspecific recombination for *Amaurobioides* using the Maximum Chisquare method in RecombiTEST [43], using only non-recombining segments for both nuclear markers (H3a and 28S).

Nucleotide compositional homogeneity within each data matrix was tested using the chi-squared metric provided in the program TreePuzzle [44], to evaluate the molecular marker’s utility for phylogenetic reconstruction at the taxonomic level intended (family level for the complete-outgroup dataset and genus level for the *Amaurobioides* dataset with two outgroup taxa). Upon confirmation that all sequences in the alignments had passed the nucleotide compositional homogeneity test, data partitioning strategies and nucleotide substitution models were selected using PartitionFinder v. 1.1.1. [45] (see Table C in S1 File for details). Furthermore, the net evolutionary distances between *Amaurobioides* species were estimated for each fragment in MEGA7 [46].

A separate phylogenetic tree was obtained for each gene fragment to evaluate gene tree discordances, after which the matrices for the gene fragments were analysed together to obtain a “concatenated” phylogeny and to estimate node ages, using the taxa for which at least 3 out of the 4 markers were available. Phylogenetic trees were obtained by Bayesian Inference (BI) in MrBayes v. 3.2.3 [47] applying a Markov Chain Monte Carlo (MCMC) simulation of 20 million generations, sampling a tree every 2,000^th^ generation for four chains in two independent runs. The data was partitioned and separate nucleotide substitution models were set as priors for the partitions based on the scheme and models selected by PartitionFinder and parameters were unlinked across partitions. Once the stationarity and correct mixing of the MCMC runs was confirmed, consensus trees were then built for each analysis using the “sumt” command discarding the first 25% as burn-in. Nodal support was evaluated through posterior probability values.

Divergence time estimates with node calibrations were carried out to obtain rates for the mitochondrial gene fragment to be used in the species tree analysis (see below), using the concatenated dataset with all outgroups, by BI with MCMC simulations in the program BEAST v. 1.8.2 [48], partitioning the data by marker, unlinking the substitution and clock model priors for each partition and setting the most appropriate substitution model (as determined by PartitionFinder) to each partition. For the tree prior a Birth-Death process was chosen (appropriate for the super-specific nature of our data) and an uncorrelated lognormal relaxed clock prior was set for each partition and the rates estimated based on a lognormal distribution around the mean (0), with an initial value and standard deviation of 1, providing a permissive range for the program to auto-optimize towards the true posterior values.

Information from two fossil anyphaenids was used to apply node age priors to the analysis. A fossil specimen in Baltic amber—estimated to be between 33.9 and 37.2 Myr old and postulated to belong to the genus *Anyphaena*—was used to set a uniform prior with a minimum age of 33.9 Myr [49]. We used this minimum age as a constraint for the mrca of anyphaenines in our study, since the assignment of this immature specimen to *Anyphaena* is not certain [50], and considering that our sampling of extant species belonging to this genus is not extensive. Information from a second fossil specimen belonging to the genus *Anyphaenoides* from Dominican amber [50] was used to set a uniform prior with a minimum age of 13.65 Myr to the mrca of *Anyphaenoides* and its sister group taxa (given the fact that only one specimen of *Anyphaenoides* was available). Two independent runs of 80 million generations each (sampling every 8,000^th^) were carried out to confirm that there was convergence. The outcomes of the two runs were also validated in Tracer v. 1.5 [51] to ensure that the effective sample sizes of the parameters were greater than 200. The tree files were then combined using LogCombiner 1.8.2 (part of the BEAST package) and the maximum clade credibility (mcc) tree with mean node heights selected in TreeAnnotator 1.8.2, setting the burn-in at 10%. Nodal support was assessed based on posterior probability, and the precision of the node age estimates were evaluated by comparing the ages to known events (the appearance of Juan Fernandez for endemic *Philisca* species, see [52] and Soto pers. obs.) and the age-calibrated rates to values found in the literature for the same molecular markers.

### Species Tree Inference

As gene-tree-species-tree discordance can present a major problem for obtaining a topology that accurately reflects the species’ evolutionary histories [53, 54], a multispecies coalescence approach was applied for *Amaurobioides*, using two outgroup taxa (*Aysenia elongata* and *Coptoprepes campanensis*). Since mitochondrial genes are presumed to be linked, we combined the COI and 16S fragments for the analyses. Species assignation was based on a combination of previous results and taxonomic status, so even though *A*. *pleta* and *A*. *pallida* were not always reciprocally monophyletic, based on the (albeit low) genetic divergence of COI (see results) and in the absence of a taxonomic revision to date, we decided to treat them as separate species.

The coalescence-based species tree analysis was carried out in BEAST v. 1.8.2 using the *BEAST (starBEAST [55]) implementation on the mitochondrial and two nuclear fragments, applying an uncorrelated lognormal relaxed clock prior to each fragment (mitochondrial, H3a and 28S), and setting the mean of the clock rate prior (ucld.mean, with a normal distribution) for the mitochondrial fragment at 0.01551 based on the geometric mean of the rates estimated by the concatenated BEAST analysis and setting the standard deviation of the normal prior at 0.3 to allow for leniency in the node-age estimation. Additionally, the node representing the mrca of *Amaurobioides* + *Aysenia elongata* was constrained and a normal distribution was chosen for the age prior, with a mean±SD of 10.9773±1.35 to obtain the same 95% confidence interval values as for the dated concatenated analysis (see Results). Nucleotide substitution models were set for each of the three partitions according to the PartitionFinder results and the tree prior was set to a Birth-Death process. Four independent MCMC chains of 100 million generations (sampling every 2,000^th^) were run, verifying the runs and selecting the maximum clade credibility tree as for the divergence dating analysis.

Due to the fact that some species in this study were represented by a single individual, and species coalescence analyses rely on several individuals per species to obtain estimates of parameters such as population size, the *BEAST analysis was re-run with the same settings and parameters as above, but removing the taxa with one individual per species (*A*. *isolata*, *A*. *litoralis* and *A*. sp. Flinders). This re-run served the purpose of verifying the extent to which these single individuals affected node age estimates. All BI runs were carried out via the CIPRES Science Gateway v. 3.3 [56].

### Tests of Monophyly

**Fig 1:**
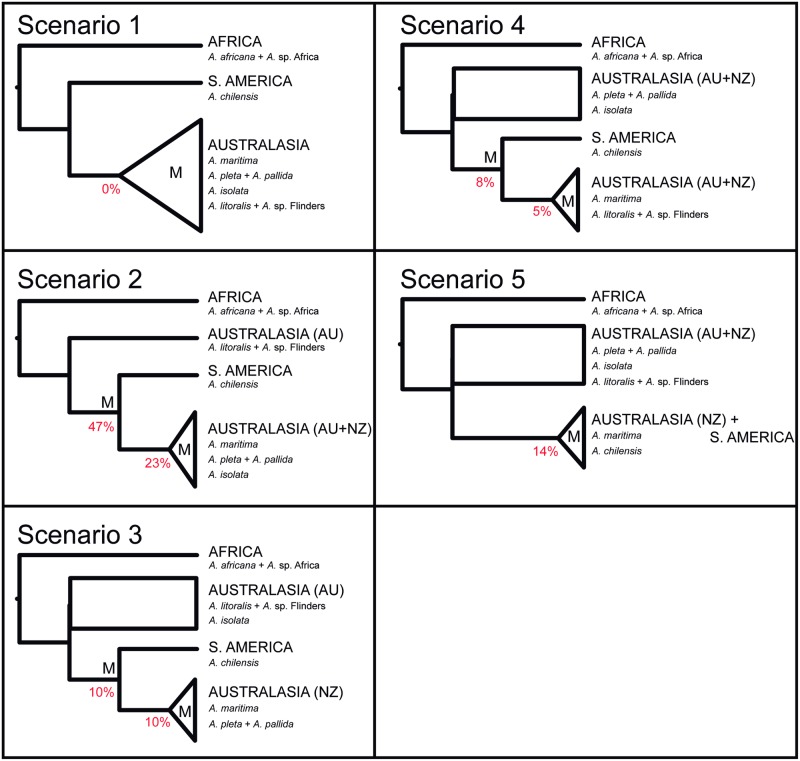
Alternative topologies for testing the phylogenetic placement of *Amaurobioides chilensis*. Schematic diagrams of five topological scenarios tested by marginal likelihood comparison obtained through steppingstone sampling in MrBayes for *Amaurobioides*, with the monophyly constrained for alternative nodes. The letter “M” indicates the nodes for which monophyly was constrained, with parsimony bootstrap percentages below. The tree terminals contain the continent in uppercase letters (with AU for Australia and NZ for New Zealand in brackets) and the species names in italics underneath. Sister species relations which were supported in the original analyses are kept as a single terminal in the schematic diagrams.

The marginal likelihoods of different hypothetical topologies were compared using steppingstone sampling [57] in MrBayes, to determine the most likely phylogenetic position of *A*. *chilensis* from South America with regards to the species from Australasia (Australia and New Zealand). Five runs with alternative constrained topologies were executed (see Fig 1), where the topologies were constrained to obtain all possible hypothetical positions of *A*. *chilensis* with regards to the species from Australasia, maintaining only the well-supported sister-group relations intact. Steppingstone sampling was set to run for 50 steps with 10 million generations. For marginal likelihood comparison, Bayes Factors were used to assess the support of the difference in log likelihoods [58] between the run with the highest marginal likelihood value and the remaining runs.

The same alternative topologies were compared using parsimony with TNT [59], with a search strategy of 100 random addition sequences, each followed by TBR (commands "mult 100;"). Tree lengths were obtained all the constrained analyses as above. The frequencies of constrained groups were also calculated in 1000 bootstrap replicates of the unconstrained dataset using the command “majority [x]” of TNT.

### Ancestral Area and Event Estimation

Ancestral ranges and speciation events for *Amaurobioides* were estimated in R version 3.2.2 [60] using the “BioGeoBEARS” v. 0.2.1 package [61, 62], which integrates and compares the Dispersal-Extinction-Cladogenesis (“DEC” [63]); DIVALIKE, modified from the DIVA program [64]; and BAYAREALIKE, modified from the BayArea program [65] algorithms, adding an extra parameter (“j”) to each method, which models “jump dispersal” or founder event speciation [66]. The following seven areas were used in the analyses: South Africa (AF), South America (AM), South Australia (AU), Tasmania (AT), southern South Island of New Zealand (NS), North Island + northern South Island of New Zealand (NN) and Antarctica (AN), to consider the possibility of Antarctica as one of the ancestral areas. The areas NS and NN were chosen based on prior information of the distributional ranges of *Amaurobioides* in New Zealand [32, 33].

Since the relative positions of the continents have not changed much during the time-frame of the analyses (see Results), a time-stratified analysis was not implemented. An area adjacency matrix was provided, setting areas as “adjacent” even when separated by the sea, but not if another area from this study was found in between (i.e. any one area could only have a maximum of three adjacent areas). Additionally, a distance matrix was built based on the distances between the furthest known points of distribution for *Amaurobioides* in each area and dividing the values by 10,000 (so that the greatest distance—10,000 km—would equate to 1 and any smaller distance would be expressed as a fraction between 0 and 1). Four different runs were executed to compare the log likelihood of different dispersal scenarios based on modifications made to the dispersal multiplier matrix. The first dispersal multiplier matrix was set as “unconstrained”, giving a probability of 1 for dispersal to occur between all areas. The second dispersal multiplier matrix was based purely on distances, subtracting the distance fraction from 1, to leave the greater distances with lower dispersal probabilities and vice-versa. The following two dispersal multiplier matrices were set to allow only dispersal from West to East (“Eastward dispersal”) or from East to West (“Westward dispersal”). All input matrices for BioGeoBEARS used in this study can be found as Matrices A-D in S1 File. Likelihoods between the scenarios and runs were compared via Bayes Factors to select the method and scenario which best explains the data.

Although the species tree was considered to provide the most reliable topology based on the aforementioned computational advantages, a second biogeographical analysis was carried out, considering the possibility of an alternative topology as inferred by the concatenated data in BEAST (and corroborated by the tests of alternative topologies), particularly regarding the sister-group relations between species from Australasia and the South American *A*. *chilensis*. The dated tree was edited in Mesquite v. 3.04 [67] to use as an input for BioGeoBEARS. The settings and priors for the ancestral range estimation analyses were set as above, except for the areas, which were reduced to four by combining the Australian and New Zealand areas into one, re-naming it Australasia. This reduction was applied because in this case the analyses were only run to corroborate the ancestral range and event estimations between Australasia and South America.

## Results

### Phylogenetics, Species Tree Inferences and Divergence Dating

**Fig 2:**
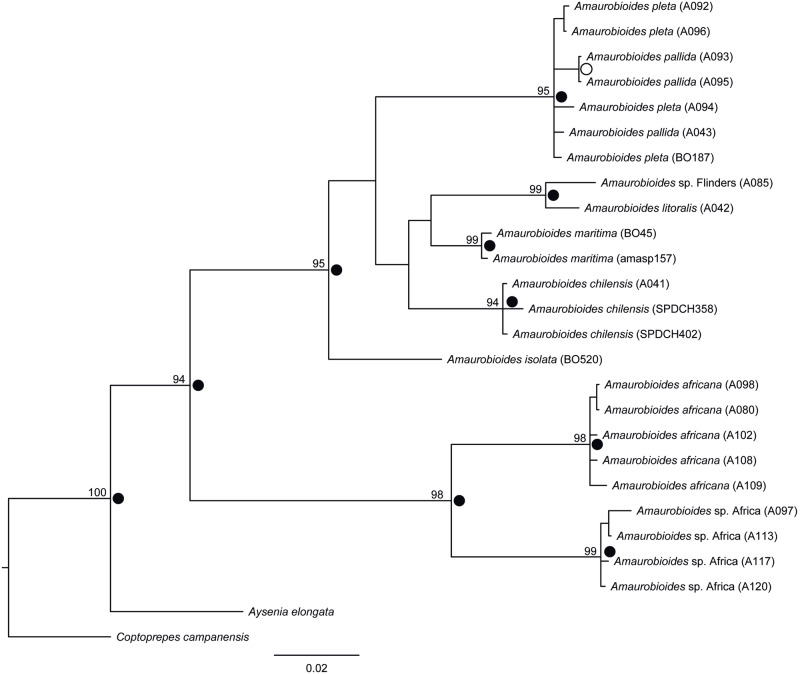
Phylogenetic tree of *Amaurobioides* inferred by MrBayes for the concatenated data. Tree inferred using COI, 16S, H3a and 28S data, obtained by 50% consensus of 10,000 trees. Bayesian posterior probabilities (PP) > = 0.9 are represented as circles at the nodes (black: 1< = PP<0.95; white: 0.95< = PP<0.9) and bootstrap support values from 1,000 replicates on the tree obtained by parsimony analysis, are shown to the left of each node. Missing values indicate the clade was not recovered.

The aligned DNA data matrices used for phylogenetic inferences contained 657, 357, 229 and 157 characters for the COI, 16S, H3a, and 28S gene fragments, respectively, after removal of hypervariable regions and recombining segments. Of these characters, the number of variable and parsimony-informative sites respectively for matrices including outgroup taxa were 292 and 245 for COI, 183 and 142 for 16S, 91 and 71 for H3a and 57 and 38 for 28S. The number of variable and parsimony-informative sites respectively for *Amaurobioides* sequences only were 139 and 118 for COI, 49 and 43 for 16S, 26 and 15 for H3a and 10 and 7 for 28S, reflecting the low evolutionary divergence based on the nuclear fragments between *Amaurobioides* species, especially those from Australia, New Zealand and South America (see Table D in S1 File). Based on the gene trees, it is also clear that the nuclear markers lack sufficient information necessary to fully resolve interspecific relationships of *Amaurobioides*, while the mitochondrial markers contain more information, sufficient for resolving the deeper nodes within the genus, including when the data is concatenated (Fig 2 and Figures A to E in S1 File).

**Fig 3:**
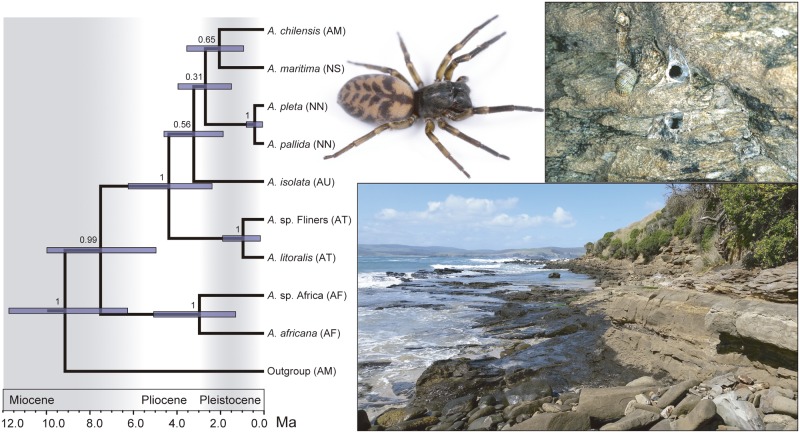
Time-calibrated species tree and photographs. Time-calibrated species tree of *Amaurobioides* using *BEAST shown on the left. Blue node bars represent 95% Highest Posterior Density intervals for node ages. Bayesian posterior probability values are shown at nodes. Codes in brackets next to terminal taxa names correspond to their areas of distribution (AM = South America; AF = Africa; AU = South Australia; AT = Tasmania; NN = North Island and northern part of South Island of New Zealand; NS = central and southern part of South Island of New Zealand). Photos to the right of the tree are of *Amaurobioides maritima* female (top left; photo M.J. Ramírez), two *A*. *maritima* retreats from Jackson Bay, South Island, New Zealand (top right; photo B.D. Opell) and typical habitat of *A*. *maritima*, Waikawa, South Island, New Zealand (bottom; photo M.J. Ramírez).

Based on the species tree coalescence analysis (Fig 3), the monophyly of *Amaurobioides* is supported, with a divergence of the most recent common ancestor (mrca) estimated between 4.95 and 9.94 Ma, 95% Highest Posterior Densities (HPD). The two species from South Africa appear to have diverged between 1.30 and 5.06 Ma, 95% HPD, and were found to be sister to the remaining species of the genus. The monophyly of the species from Australia, New Zealand and South America is also supported, with the mrca diverging approximately 4.38 Ma (95% HPD: 2.30–6.22 Ma), yet species relations within this clade are generally poorly supported. The only sister group relations supported within this group are *A*. *pallida* + *A*. *pleta* (from New Zealand) and *A*. *litoralis* + *Amaurobioides* sp. from Flinders Island (both from Tasmania), estimated to have diverged 0.96 and 0.43 Ma, respectively. Removing the species with a single representative from the analyses had no major effect on node age estimates, as can be seen from the 95% HPD for both trees (see Figure F in S1 File).

The marginal likelihoods (in log units) for the tests of alternative topologies within the Australasia+South America clade can be found in Table 1. Taking into account that the support for the hypothesis with the higher log likelihood value is considered positive if twice the difference in log likelihoods (i.e. the Bayes Factor) is greater than 2 [57], the most likely scenario with positive to strong support in this case is that the species from South America falls within the clades from Australasia (as for the concatenated analyses, Fig 2 and Figure E in S1 File). The least likely scenario would be *A*. *chilensis* from South America as sister to a clade from Australasia (scenario 1). Similarly, in the parsimony analysis the alternative resolution with *A*. *chilensis* as sister to the Australasian species is the least parsimonious (see Table 1), and never appears in the bootstrap pseudoreplicates (Fig 1), whereas scenario 2 received the lowest tree-length value and of all the scenarios is therefore considered the most parsimonious.

**Table 1:** Marginal likelihoods in log-units (lnL), Bayes Factors (BF) and parsimony tree lengths (PL) for the alternative topological scenarios tested for *Amaurobioides* as depicted in Fig 1, obtained by steppingstone sampling in MrBayes and topological comparisons in TNT. Asterisks indicate the scenario which received the highest log likelihood value.

Scenario	lnL	BF	PL
Scenario 1	-3065.65	13.62	426
Scenario 2	-3061.52	5.36	419
Scenario 3	-3062.18	6.68	424
Scenario 4	-3058.84	***	424
Scenario 5	-3065.21	12.74	421

The node age estimates obtained from the concatenated matrices were generally older, which has been noted and justified previously [68]. The divergence of the mrca of *Amaurobioides* was estimated at 10.08 million years ago (Ma; 7.52–12.58, 95% HPD; Figure E in S1 File). Based on these node age estimates, the species from South Africa appear to have diverged around 5.79 Ma, while the sister group containing the species from Australia, New Zealand and South America began diversifying around 6.44 Ma. The split between *A*. *pleta* and *A*. *pallida* was estimated at around 1.03 Ma, and between *A*. *litoralis* and *Amaurobioides* sp. from Flinders Island around 1.28 Ma. Based on this tree, the relations between the Australian, New Zealand and South American taxa are not fully resolved either. The mean rates obtained for the four markers used in this study are comparable to molecular rates obtained for the same markers from different studies (see Table E in S1 File). Furthermore, the divergence of the *Philisca* species endemic to Juan Fernandez Island was estimated at 1.59 Ma (1.03–2.18, 95% HPD), therefore younger than the island itself (consistent with the volcanic origin of the island 4 Ma), as expected (Soto pers. obs.). We therefore consider the node age estimates for *Amaurobioides* reliable for the purpose of this study.

### Ancestral Area and Event Estimation

**Fig 4:**
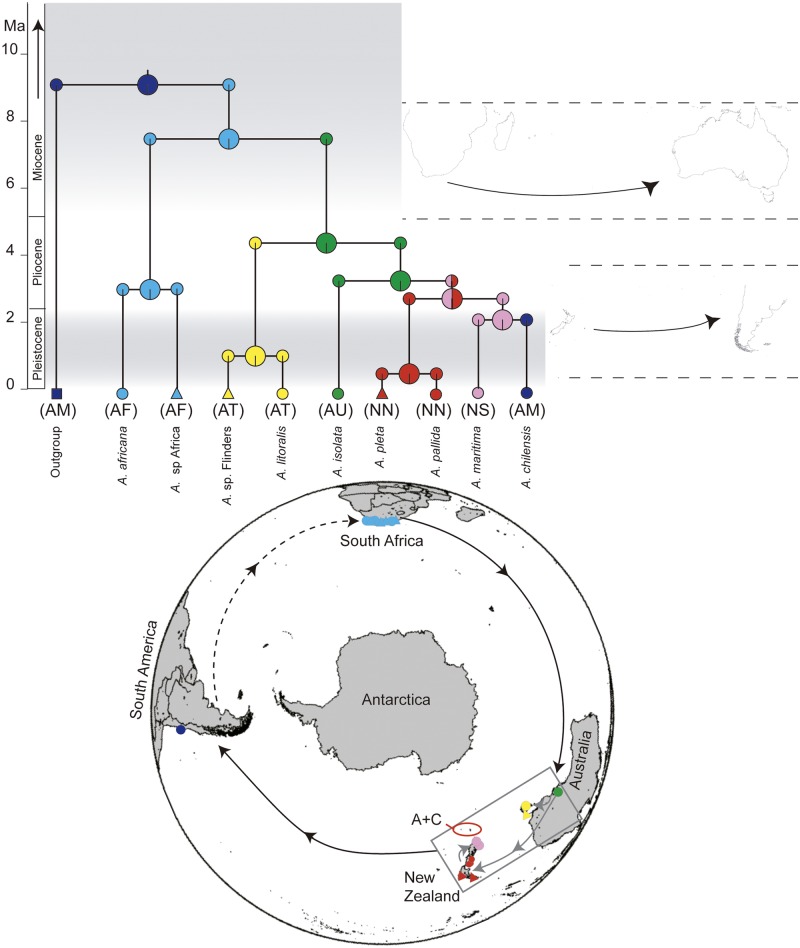
Biogeographical areas and events estimations. Biogeographical areas and events estimations obtained from the West Wind Drift-constrained run with the DIVALIKE+J algorithm. Coloured symbols at the tips of the species tree represent the current sampling localities of the specimens used in this study, shown also on the bottom map using the same colours and shapes. Additionally, codes in brackets by the tips of the species tree correspond to the areas of distribution (AM = South America; AF = Africa; AU = South Australia; AT = Tasmania; NN = North Island and northern part of South Island of New Zealand; NS = central and southern part of South Island of New Zealand). Pie charts at the nodes of the species tree represent the relative probabilities of the ancestral areas, while pie charts at the corners represent the relative probabilities of founder-effect dispersal to new areas. Smaller maps to the right of the species tree represent historical events during given epochs, with arrows representing estimated long-distance dispersal events. Bottom map contains the sampling localities of the specimens used in this study, colour and shape coded as in the terminal branches of the tree. Arrows represent dispersal events inferred in this study (solid line: ancestral *Amaurobioides* species; dashed line: mrca of *Amaurobioides*). The grey box around Australia and New Zealand indicates that the exact events within those areas remain unresolved. The red oval marked A+C shows the position of Auckland and Campbell Islands.

The BioGeoBEARS run, using the species tree, with the highest log likelihood values for most models was the “Eastward dispersal” run, with the dispersal multiplier matrix set to favour dispersal from the West to the East and within each run. Additionally, the DIVALIKE+J model was always favoured over the others (see Table F in S1 File), although the difference in log likelihoods with the BAYAREALIKE+J run was minimal. Based on the DIVALIKE+J outcome for the “Eastward dispersal” run, the ancestral area of *Amaurobioides* was Africa. Since the remaining genera of Anyphaeninae (outgroups to *Amaurobioides* in this study) are distributed in South America, a dispersal event from South America to Africa during the Miocene is likely to have established the ancestral populations of *Amaurobioides* (see Fig 4). Founder-effect type events following long-distance dispersal from Africa to Australia during the late Miocene most likely established the ancestral area of the Australia + New Zealand + South America clade. Since the phylogenetic relationships within this group were not resolved, most biogeographic event estimations for this clade cannot be commented on with certainty. However, even based on the most likely BioGeoBEARS run (Table G in S1 File) using the alternative topology from the concatenated data, ancestral populations of *A*. *chilensis* were established in South America after a long-distance dispersal event from Australasia (Figure G in S1 File). Furthermore, the re-colonisation of South America from Australasia is highly likely based on several lines of evidence discussed below.

## Discussion

### Phylogenetic Inferences

In this study the relationships between *Amaurobioides* species were only fully resolved at a deeper level and in some derived sister group relationships. The poor resolution within the Australian and New Zealand clade is probably due to the low variability, i.e. phylogenetic signal, of the nuclear markers. A recent phylogeographic study [26] disentangles the relationships between Australian and New Zealand species using denser sampling and different molecular markers. Adding more taxa and especially more (rapidly-evolving) molecular markers may provide a clearer picture of the evolutionary processes that have shaped the current diversity in said areas. The coastal areas between South Australia (where the *A*. *isolata* specimen was collected) and south-eastern Australia, just north of Tasmania and Flinders Island, appear promising for more *Amaurobioides* diversity still to be discovered. Perhaps this missing information may explain some of the low support for internal branches of the phylogenies, which would be better resolved with a broader sampling from Australia.

Regarding the *Amaurobioides* species from New Zealand, even though there are eight described species to date, their validity is questionable [26, 32, 33]. With this in mind, the species used in this study represent the genetic diversity found on the North and South Islands of New Zealand. The only valuable addition would therefore be *A*. *piscator* from New Zealand’s Sub-Antarctic Auckland and Campbell islands [25, 30, 33]. Future systematic contributions will include a revision of New Zealands’ *Amaurobioides* as well as formal descriptions of the undescribed species used in this study.

### Historical Biogeography

The ancestral area of the genus *Amaurobioides* was estimated to be southern Africa, upon the establishment of (an) ancestral population(s) during the Miocene from South America, consistent with the beginning of the Arctic Circumpolar Current [5]. Since the Gondwanan continents had separated by the Miocene, the ancestral population that gave rise to the mrca of *Amaurobioides* is likely to have arrived to Africa from the west by long-distance dispersal. The relatively deep divergence between the species found in South Africa are further evidence supporting the long time span of this genus’ presence in the continent, and therefore its plausibility as the ancestral area. South Africa, in particular the Cape area, is well-known for its extremely high level of species richness and endemism of plants [69], which have undergone numerous long-distance dispersal events to and from South Africa [9]. Apart from plants, the African continent is the ancestral area, and the source of long-distance dispersal events, for other organisms, including nymphalid butterflies [70], thrushes [71], platyrrhine primates [72], and with the findings of the present study, the genus *Amaurobioides* as well.

Further eastward long-distance dispersal events of *Amaurobioides* from Africa to Australasia (Australia and New Zealand) around the Mio-Pliocene boundary and the mid-Pliocene were estimated in this study, establishing present-day species in South Australia, Tasmania, North Island and South Island of New Zealand. Nevertheless, the aforementioned sampling gap from Australia’s south-eastern coast and the absence of *A*. *piscator* may not only have weakened nodal support, but also obscured other possible events regarding *Amaurobioides* in said areas during the Pliocene. Alternatively, the low nodal support may not be a cause for diluted biogeographical signal, but an effect of it, if repeated dispersal between Australia and New Zealand gave rise to prolonged periods of genetic introgression and hybridisation, as may be expected between adjacent landmasses [7]. As populations then established and speciated, it is likely that secondary colonisation became more complicated [6, 73]. The divergence of the mrca from Australia and New Zealand, estimated at around 4.36 Ma is similar to previously suggested ranges (4.0–4.6 Ma [32]; 4.497 Ma: mrca of Australian + NZ species [26]). While it is still unclear whether the species from New Zealand are monophyletic (this study’s species tree, [32, 33] or not [26], their biogeographical history remains to be thoroughly tested.

The postulated Plio-Pleistocene boundary colonisation of South America from New Zealand by *Amaurobioides* found in this study in a sense closes the circle of long-distance dispersal followed by colonisation events and speciation of this genus. Although the sister group relation of *A*. *chilensis* and *A*. *maritima* was not supported by molecular evidence in this study, the topological tests allow us to rule out a monophyletic group from Australasia and therefore lend support to an Australasian origin for the South American species. In addition, morphological evidence lends support to the sister group relation of *A*. *chilensis* and *A*. *maritima*, as the females of the two species are virtually indistinguishable [29]. Furthermore, since *Amaurobioides* is only distributed along the west coast of South America, its colonisation from the east is more likely than colonisation, range expansion and local extinction from the eastern or southern parts of South America. Specimens of *A*. *chilensis* have been found in localities along the Chilean coast at least 1200 km apart [31]. A more in-depth phylogeographic study of *A*. *chilensis* would therefore be necessary to trace its colonisation and expansion history, as well as estimating the species’ genetic diversity since the early Pleistocene.

Other possible explanations for *Amaurobioides* species’ dispersal routes between continents are less plausible and would require further evidence. For example, the role of Antarctica in the genus’ biogeographical history is difficult to conceive, due to its coasts being currently uninhabitable for *Amaurobioides*. However, it may be that during warmer periods of the earth’s history (late Miocene/early Pliocene), Antarctica may also have harboured ancestral populations, presenting an additional geographic range for dispersal to- and from. Including *A*. *piscator* in the analyses may shed light on more southern events, in that we could infer the direction from which they reached Auckland and Campbell Islands, although considering the geographic position of these islands, they are approximately five times closer to New Zealand than to Antarctica and therefore the spiders are more likely to have arrived from New Zealand. In any case, only fossil evidence from Antarctica itself would unambiguously lend support to the presence of past populations or species on the continent, since even the likelihood-based biogeographical analyses of this study never estimated Antarctica as an ancestral area.

Despite a few uncertainties in the phylogenetic results, the overwhelming patterns found in this study support repeated eastward long-distance dispersal events by ancestral *Amaurobioides* populations acting as founder events in distant coastlines of other continents. This eastward trend fits in with predictions based on the Antarctic Circumpolar Current and the West Wind Drift. The West Wind Drift has been used to explain the distribution of wind-dispersed organisms in the Southern Hemisphere, particularly plants [8, 9]. Unlike many widely distributed genera of small spiders, well-known to disperse by aerial ballooning [74], we find it unlikely that *Amaurobioides* disperse by that mechanism. It has been shown that species that are specialists of fragmented habitats have a low propensity for ballooning [75], and *Amaurobioides* use a narrow, highly fragmented niche, where almost any wind would take them away to the water, land, or unsuitable sandy beaches. Other circumstantial evidence supports this reasoning. *Amaurobioides* specimens were collected by probing them out of their cells with a metal wire; as the spiders are forced out they gasp firmly on the rock surface, run or hide, but never jump (MJR, BDO, CRH, pers, obs.). This behavior is in contrast with the usual escape strategy of most entelegyne spiders and anyphaenids in general, of jumping away or dropping while leaving a security dragline [10]. With such reluctance to lose grasp of a solid substrate, we find it much less likely that they would adventure in ballooning.

The other alternative to explain transoceanic long-distance dispersal of this genus would therefore be oceanic drift, or rafting, aided by the Antarctic Circumpolar Current and possibly the West Wind Drift pushing floating matter along the ocean’s surface, as found for other organisms, e.g. [76–80]. Relatively few studies have postulated [20–24, 26] or even observed [19, 81] rafting in low-vagility spiders. While there are myriads of spider genera endemic to high latitudes in the Southern Hemisphere, and many of them belong to excellent ballooning families, *Amaurobioides* is, to our knowledge, the only one that inhabits South Africa, Australia, New Zealand and South America [24, 82]. Another genus with a similar geographical pattern is *Desis* (Desidae), but it extends into lower latitudes (Africa, southern India, South East Asia and Japan, islands of the South Pacific Ocean including Australia and New Zealand, and the Galapagos Islands). Both genera inhabit coastal zones and we believe that rafting is a more plausible mechanism than ballooning to explain their distribution, agreeing with the hypothesis presented by Hewitt [35]. This hypothesis is strengthened by the current findings on *Amaurobioides* mirroring the patterns found in marine fauna also believed to have distributed by rafting [83], their coastal habits, and proved ability to withstand immersion [29, 84]. This study therefore represents an exceptional case for spiders of a Southern Hemisphere distribution shaped by successful Long Distance Dispersal events by founder individuals/ populations rafting with the Antarctic Circumpolar Current and the West Wind Drift.

This work adds to the literature showing that *Amaurobioides* is a remarkable anyphaenid spider. Being the only genus of the sub-family with species found outside the American continent, its adaptation to living in coastal habitats allowed for transoceanic dispersal events, presumably by rafting, to establish new species on the coasts of Southern Africa, Australia, New Zealand and South America. Furthermore, the dispersal events are likely to have been in a predominantly eastward direction, fitting in timing and direction with the onset and continued geo-climatic phenomena of the Antarctic Circumpolar Current and West Wind Drift. This study therefore adds to the cases of dispersal asymmetry found in the Southern Hemisphere [6–9, 85], not only for well-known long-distance dispersers but also for organisms with poor dispersal abilities of their own, such as *Amaurobioides*.

## Supporting Information

S1 File **Table A.** Information for tissue samples and GenBank accession codes for sequences. **Table B.** Primers used for PCR. **Table C.** Partitioning scheme and nucleotide substitution models for phylogenetic analyses. **Table D.** Estimates of net evolutionary divergence. **Table E.** Mean rates for molecular markers. **Table F.** BioGeoBEARS outputs based on the *BEAST species tree. **Table G.** BioGeoBEARS outputs based on the concatenated BEAST tree. **Matrix A.** Unconstrained dispersal multiplier matrix. **Matrix B.** Distance constrained dispersal multiplier matrix. **Matrix C.** East-to-west (EWD) constrained dispersal multiplier matrix. **Matrix D.** West-to-east (WWD) constrained dispersal multiplier matrix. **Figure A.** Phylogenetic gene tree for COI. **Figure B.** Phylogenetic gene tree for 16S. **Figure C.** Phylogenetic gene tree for H3a. **Figure D.** Phylogenetic gene tree for 28S. **Figure E.** Chronogram inferred using the concatenated COI, 16S, H3a and 28S data. **Figure F.** Species coalescence tree with node age estimates based COI, 16S, H3a and 28S. **Figure G.** Ancestral range estimates based on the concatenated topology from BEAST.
(DOC)
