# Supplementary material for: Around the World in Eight Million Years: Historical Biogeography and Evolution of the Spray Zone Spider Amaurobioides (Araneae: Anyphaenidae)
Source: PLoS One. 2016 Oct 12;11(10):e0163740. doi: 10.1371/journal.pone.0163740 (PMC5061358; doi:10.1371/journal.pone.0163740)
Supplement: S1 File — Table A. Information for tissue samples and GenBank accession codes for sequences. Table B. Primers used for PCR. Table C. Partitioning scheme and nucleotide substitution models for phylogenetic analyses. Table D. Estimates of net evolutionary divergence. Table E. Mean rates for molecular markers. Table F. BioGeoBEARS outputs based on the *BEAST species tree. Table G. BioGeoBEARS outputs based on the concatenated BEAST tree. Matrix A. Unconstrained dispersal multiplier matrix. Matrix B. Distance constrained dispersal multiplier matrix. Matrix C. East-to-west (EWD) constrained dispersal multiplier matrix. Matrix D. West-to-east (WWD) constrained dispersal multiplier matrix. Figure A. Phylogenetic gene tree for COI. Figure B. Phylogenetic gene tree for 16S. Figure C. Phylogenetic gene tree for H3a. Figure D. Phylogenetic gene tree for 28S. Figure E. Chronogram inferred using the concatenated COI, 16S, H3a and 28S data. Figure F. Species coalescence tree with node age estimates based COI, 16S, H3a and 28S. Figure G. Ancestral range estimates based on the concatenated topology from BEAST. (DOC) [file pone.0163740.s001.doc]

**Supporting Information File S1**

**Around the World in Eight Million Years: historical biogeography and evolution of the spray zone spider *Amaurobioides* (Araneae: Anyphaenidae)**

F.S. Ceccarelli, B.D. Opell, C.R. Haddad, R.J. Raven, E.M. Soto, M.J. Ramírez

**Table A.** Numbers and provenance data for tissue samples from which DNA was extracted, and GenBank accession codes for cytochrome c oxidase subunit I (COI), 16S rDNA (16S), 28S rDNA (28S), and Histone 3-a (H3a) sequences used in phylogenetic and biogeographical analyses of *Amaurobioides* and outgroup taxa. Accession codes in **bold** typeface are for sequences generated *de novo* for this study.

|  |  | |  |  |  |  |  |  | Genbank accession numbers | | | |
| --- | --- | --- | --- | --- | --- | --- | --- | --- | --- | --- | --- | --- |
|  | Genus | | species | DNA code | Specimen code | Country | Latitude | Longitude | COI | 16S | H3a | 28S |
| Anyphaenidae, Anyphaenidae | | | | | | | | | | | | |
|  | *Amaurobioides* | | sp. Flinders | A082 | QM R. Raven | Australia | -40.228996 | 148.027816 | **KX817459** | **-** | **-** | **-** |
|  | *Amaurobioides* | | sp. Flinders | A083 | QM R. Raven | Australia | -40.228996 | 148.027816 | **KX817460** | **-** | **-** | **-** |
|  | *Amaurobioides* | | sp. Flinders | A084 | QM R. Raven | Australia | -40.228996 | 148.027816 | **KX817461** | **-** | **-** | **-** |
|  | *Amaurobioides* | | sp. Flinders | A085 | QM R. Raven | Australia | -40.228996 | 148.027816 | **KX817462** | **KX817385** | **KX817509** | **KX817425** |
|  | *Amaurobioides* | | *isolata* | BO520 | BO520 | Australia | -34.86272 | 135.71988 | **KX817463** | **KX817386** | - | **KX817426** |
|  | *Amaurobioides* | | *litoralis* | A042 | MACN-Ar 30579 | Australia | -43.00915 | 147.93235 | **KX817464** | **KX817387** | **KX817510** | **KX817427** |
|  | *Amaurobioides* | | *chilensis* | A041 | MACN-Ar 28555 | Chile | -30.07032 | -71.37633 | **KX817465** | **KX817388** | **KX817511** | **KX817428** |
|  | *Amaurobioides* | | *chilensis* | SPDCH358 | MACN-Ar 28561 | Chile | -30.07032 | -71.37633 | **KX817507** | **-** | **-** | **-** |
|  | *Amaurobioides* | | *chilensis* | SPDCH402 | MACN-Ar 28556 | Chile | -30.07032 | -71.37633 | **KX817508** | **-** | **-** | **-** |
|  | *Amaurobioides* | | *pallida* | A043 | MLB 04033 | New Zealand | -41.326142 | 174.869706 | - | **KX817389** | - | - |
|  | *Amaurobioides* | | *pleta* | A094 | BO316 | New Zealand | -37.938933 | 177.013283 | **KX817466** | **KX817390** | - | **KX817429** |
|  | *Amaurobioides* | | *pleta* | A096 | BO390 | New Zealand | -36.302183 | 174.796383 | **KX817467** | **KX817391** | - | **KX817430** |
|  | *Amaurobioides* | | *pleta* | BO187 | BO187 | New Zealand | -35.562033 | 174.492433 | - | **KX817392** | **KX817512** | **KX817431** |
|  | *Amaurobioides* | | *pleta* | A092 | BO184 | New Zealand | -35.562033 | 174.492433 | **KX817468** | **KX817393** | **KX817513** | - |
|  | *Amaurobioides* | | *pallida* | A095 | BO361 | New Zealand | -41.342717 | 174.79155 | **KX817469** | **KX817394** | **KX817514** | **KX817432** |
|  | *Amaurobioides* | | *maritima* | amasp157 | CASENT-9021414 | New Zealand | -46.48433 | 169.71068 | KR558954 | KR558837 | KR558771 | KR558888 |
|  | *Amaurobioides* | | *maritima* | BO45 | BO45 | New Zealand | -46.61165 | 168.359867 | **KX817470** | **KX817395** | **KX817515** | **KX817433** |
|  | *Amaurobioides* | | *pallida* | A093 | BO258 | New Zealand | -41.0503 | 173.018783 | **KX817471** | **KX817396** | **KX817516** | **KX817434** |
|  | *Amaurobioides* | | *africana* | A080 | CASENT-9023621 | South Africa | -34.140551 | 18.321588 | **KX817472** | **KX817397** | **KX817517** | **KX817435** |
|  | *Amaurobioides* | | sp. Africa | A097 | SAM-ENW_C006529 | South Africa | -33.153244 | 27.699237 | **KX817473** | **KX817398** | **KX817518** | **KX817436** |
|  | *Amaurobioides* | | *africana* | A098 | SAM-ENW_C006635 | South Africa | -34.140067 | 18.3209 | **KX817474** | **KX817399** | **KX817519** | **KX817437** |
|  | *Amaurobioides* | | sp. Africa | A099 | HaddadWiese | South Africa | -34.019433 | 24.928817 | **KX817475** | - | **KX817520** | - |
|  | *Amaurobioides* | | *africana* | A100 | SAM-ENW_C006546 | South Africa | -34.37083 | 18.88 | **KX817476** | - | **KX817521** | - |
|  | *Amaurobioides* | | *africana* | A101 | SAM-ENW_C006920 | South Africa | -34.340556 | 18.472778 | **KX817477** | - | **KX817522** | - |
|  | *Amaurobioides* | | *africana* | A102 | SAM-ENW_C006953 | South Africa | -34.162222 | 18.431944 | - | **KX817400** | **KX817523** | **KX817438** |
|  | *Amaurobioides* | | *africana* | A103 | SAM-ENW_C006919 | South Africa | -34.162222 | 18.431944 | **KX817478** | - | **KX817524** | - |
|  | *Amaurobioides* | | sp. Africa | A104 | SAM-ENW_C006531 | South Africa | -33.98333 | 25.67139 | **KX817479** | - | - | - |
|  | *Amaurobioides* | | *africana* | A105 | SAM-ENW_C006950 | South Africa | -34.140556 | 18.320556 | **KX817480** | - | **KX817525** | - |
|  | *Amaurobioides* | | *africana* | A106 | SAM-ENW_C006923 | South Africa | -34.340556 | 18.472778 | **KX817481** | - | **KX817526** | - |
|  | *Amaurobioides* | | *africana* | A107 | SAM-ENW_C006927 | South Africa | -34.340556 | 18.472778 | - | - | **KX817527** | - |
|  | *Amaurobioides* | | *africana* | A108 | SAM-ENW_C006547 | South Africa | -34.07444 | 23.01944 | - | **KX817401** | **KX817528** | **KX817439** |
|  | *Amaurobioides* | | *africana* | A109 | SAM-ENW_C006937 | South Africa | -34.140556 | 18.320556 | - | **KX817402** | **KX817529** | **KX817440** |
|  | *Amaurobioides* | | *africana* | A110 | SAM-ENW_C006634 | South Africa | -34.357483 | 18.473833 | **KX817482** | - | **KX817530** | - |
|  | *Amaurobioides* | | *africana* | A111 | SAM-ENW_C006955 | South Africa | -33.692433 | 26.673217 | **KX817483** | - | **KX817531** | - |
|  | *Amaurobioides* | | *africana* | A112 | SAM-ENW_C006532 | South Africa | -34.42694 | 21.34167 | - | - | **KX817532** | - |
|  | *Amaurobioides* | | sp. Africa | A113 | SAM-ENW_C006543 | South Africa | -34.17417 | 22.13583 | **KX817484** | **KX817403** | **KX817533** | **KX817441** |
|  | *Amaurobioides* | | sp. Africa | A114 | SAM-ENW_C006537 | South Africa | -33.98222 | 23.57611 | **KX817485** | - | **KX817534** | - |
|  | *Amaurobioides* | | *africana* | A115 | SAM-ENW_C006922 | South Africa | -34.340556 | 18.472778 | **KX817486** | - | **KX817535** | - |
|  | *Amaurobioides* | | *africana* | A116 | SAM-ENW_C006544 | South Africa | -34.67056 | 19.50056 | - | - | **KX817536** | - |
|  | *Amaurobioides* | | *africana* | A117 | SAM-ENW_C006921 | South Africa | -34.162222 | 18.431944 | - | **KX817404** | **KX817537** | **KX817442** |
|  | *Amaurobioides* | | *africana* | A118 | SAM-ENW_C006924 | South Africa | -34.340556 | 18.472778 | - | - | **KX817538** | - |
|  | *Amaurobioides* | | *africana* | A119 | SAM-ENW_C006939 | South Africa | -34.140556 | 18.320556 | **KX817487** | - | **KX817539** | - |
|  | *Amaurobioides* | | sp. Africa | A120 | SAM-ENW_C006545 | South Africa | -34.82472 | 20.02722 | **KX817488** | **KX817405** | **KX817540** | **KX817443** |
|  | *Amaurobioides* | | sp. Africa | A122 | SAM-ENW_C006926 | South Africa | -34.162222 | 18.431944 | **KX817489** | - | **KX817541** | - |
|  | *Amaurobioides* | | *africana* | amaaf099 | - | South Africa | - | - | - | - | **KX817542** | **KX817444** |
|  | *Acanthoceto* | | *acupicta* | acaac031 | MACN-Ar-12068 | Argentina | -37.31781 | -59.150391 | KR558950 | KR558833 | KR558767 | KR558884 |
|  | *Acanthoceto* | | *cinereus* | acaci096 | MACN-Ar-30585 | Argentina | -40.855583 | 71.612222 | KR558951 | KR558834 | KR558768 | KR558885 |
|  | *Acanthoceto* | | *ladormida* | A036 | MACN-Ar-28560 | Chile | -32.143784 | -71,533335 | **KX817490** | **KX817406** | **KX817543** | **KX817445** |
|  | *Acanthoceto* | | *marina* | E73 | MACN-Ar-25792 | Chile | -36.8065 | -73.176528 | KR558952 | KR558835 | KR558769 | KR558886 |
|  | *Acanthoceto* | | *pichi* | E67 | MACN-Ar-28390 | Argentina | -41.51625 | -71,53567 | KR558953 | KR558836 | KR558770 | KR558887 |
|  | *Acanthoceto* | | *riogrande* | A039 | MJR-1538 | Argentina | -40.726472 | -64.967613 | **KX817491** | **KX817407** | **KX817544** | **KX817446** |
|  | *Arachosia* | | *praesignis* | arapa029 | MACN-Ar-12067 | Argentina | -37.31781 | -59.150391 | KR558957 | KR558840 | KR558774 | KR558891 |
|  | *Araiya* | | *coccinea* | aryco077/A046 | MACN-Ar-31157 | Chile | -42.6182 | -74.101216 | KR558959 | **KX817408** | KR558776 | KR558893 |
|  | *Axyracrus* | | *elegans* | A048 | MACN-Ar-31154 | Argentina | -55.584 | -65.1335 | **KX817492** | **KX817409** | **KX817545** | **KX817447** |
|  | *Aysenia* | | *cylindrica* | A089 | CASENT-9034109 | Chile | -39.713 | -73.356 | **KX817493** | **KX817410** | **KX817546** | **KX817448** |
|  | *Aysenia* | | *elongata* | ayel009 | MACN-Ar-10869 | Chile | -37.8275 | -73.008944 | KR558961 | - | KR558778 | KR558895 |
|  | *Aysenia* | | *izquierdoi* | ayasp072 | CASENT-9029773 | Chile | -40.0075 | -73.646389 | KR558962 | KR558842 | KR558779 | KR558896 |
|  | *Aysenoides* | | *colecole* | aysco78 | CASENT-9029782 | Chile | -42.617817 | -74.099183 | KR558964 | - | KR558781 | KR558898 |
|  | *Aysenoides* | | *nahuel* | aysna076 | CASENT-9029770 | Chile | -39.994783 | -73.587117 | KR558965 | KR558844 | KR558782 | KR558899 |
|  | *Aysenoides* | | *parvus* | ayspa003/A049 | MACN-Ar-28845 | Chile | -41.509944 | -72.612389 | KR558966 | **KX817411** | KR558783 | KR558900 |
|  | *Aysenoides* | | *simoi* | A077 | CASENT-9039001 | Chile | -38.44 | -71.509 | **KX817494** | **KX817412** | **KX817547** | **KX817449** |
|  | *Coptoprepes* | | *campanensis* | copca002 | MACN-Ar-28847 | Chile | -32.942611 | -71.083917 | KR558968 | KR558846 | KR558785 | KR558902 |
|  | *Coptoprepes* | | *flavopilosus* | A050 | MACN-Ar-29569 | Argentina | -54.88383 | -67.21405 | **KX817495** | **KX817413** | **KX817548** | **KX817450** |
|  | *Coptoprepes* | | *valdiviensis* | copvaE62 | CASENT-9027973 | Chile | -41.4672 | -72.649933 | KR558969 | KR558847 | KR558786 | KR558903 |
|  | *Ferrieria* | | *echinata* | ferec034 | MACN-Ar-10875 | Chile | -37.8275 | -73.008944 | KR558970 | KR558848 | KR558787 | KR558904 |
|  | *Gamakia* | | *hirsuta* | gamhi023 | MACN-Ar-28782 | Chile | -36.7045 | -71.60267 | KR558971 | - | KR558788 | KR558905 |
|  | *Gayenna* | | *americana* | gayam213/230 | MACN-Ar-28843 | Chile | -37.8275 | -73.008944 | KR558972 | KR558849 | KR558789 | KR558906 |
|  | *Gayennoides* | | *molles* | E72 | MACN-Ar-25795 | Chile | -28.335555 | -71.161805 | KR558973 | KR558850 | KR558790 | KR558907 |
|  | *Josa* | | *calilegua* | A072 | MACN-Ar 25841 | Argentina | -23.6811 | -64.9014 | **KX817496** | **KX817414** | **KX817549** | **KX817451** |
|  | *Josa* | | *riveti* | E75 | MACN-Ar 28546 | Ecuador | -0.61407 | -78.47341 | KR558976 | - | KR558793 | KR558909 |
|  | *Josa* | | *spBolivia* | A078 | MACN-Ar 31168 | Bolivia | -16.39488 | -67.55847 | **KX817497** | **KX817415** | **KX817550** | **KX817452** |
|  | *Monapia* | | *dilaticollis* | mondi170/A130 | MACN-Ar 29170 | Chile | -35.8338 | -72.510217 | KR558978 | **KX817416** | KR558795 | KR558911 |
|  | *Negayan* | | *excepta* | A017 | MACN-Ar 29509 | Argentina | -54.870215 | -67.340163 | **KX817498** | **KX817417** | **KX817551** | **KX817453** |
|  | *Negayan* | | *paduana* | negpa062 | ARAMR000062 | Chile | -39.356945 | -71.967778 | KR559013 | KR558882 | KR558830 | KR558946 |
|  | *Negayan* | | *puno* | A124 | MACN-Ar-34632 | Argentina | -31.62392 | -69.25446 | **KX817499** | **KX817418** | **KX817552** | **KX817454** |
|  | *Oxysoma* | | *punctatum* | Oxypu060/A059 | ARAMR000060 | Chile | -40.666389 | -72.171945 | **KX817500** | **KX817419** | KR558832 | KR558949 |
|  | *Phidyle* | | *punctipes* | phypu024 | MACN-Ar-28783 | Chile | -32.016806 | -71.508056 | KR558997 | KR558867 | KR558813 | KR558929 |
|  | *Philisca* | | *accentifera* | E01 | MACN-Ar 28097 | Chile | -32.54593 | -71.45179 | KX817663 | KX817558 | KX817764 | KX817612 |
|  | *Philisca* | | *amoena* | phiamE19 | CASENT-9039109 | Chile | -39.701183 | -73.309117 | KR558988 | KR558858 | KR558804 | KR558920 |
|  | *Philisca* | | *atrata* | E22 | MACN-Ar-28020 | Argentina | -41.51625 | -71.53567 | KR558993 | KR558863 | KR558809 | KR558925 |
|  | *Philisca* | | *doilu* | phido014 | MACN-Ar-10934 | Chile | -39.461694 | -71.845611 | KR558989 | KR558859 | KR558805 | KR558921 |
|  | *Philisca* | | *hahni* | A061 | MACN-Ar-29576 | Argentina | -54.88383 | -67.21405 | **KX817501** | - | **KX817553** | **KX817455** |
|  | *Philisca* | | *huapi* | E47 | MACN-Ar 28150 | Chile | -38.01283 | -73.18761 | KX817699 | KX817606 | KX817746 | KX817612 |
|  | *Philisca* | | *hyadesi* | F26/E13 | MACN-Ar-30584 | Chile | -39.138528 | -71.708583 | KR558991 | KR558861 | KR558807 | KR558923 |
|  | *Philisca* | | *ingens* | E51 | MACN-Ar 27955 | Chile | -33.65276 | -78.84403 | KX817703 | KX817571 | KX817714 | KX817651 |
|  | *Philisca* | | *ornata* | E34 | MACN-Ar 28062 | Chile | -33.63087 | -78.84386 | KX817687 | KX817576 | KX817726 | KX817635 |
|  | *Philisca* | | *pizarroi* | E10 | MACN-Ar 28042 | Chile | -33.63790 | -78.85302 | KX817671 | KX817586 | KX817720 | KX817619 |
|  | *Philisca* | | *robinson* | E49 | MACN-Ar 27962 | Chile | -33.65276 | -78.84403 | KX817701 | KX817583 | KX817741 | KX817649 |
|  | *Philisca* | | *sp5* | E55 | MACN-Ar 28084 | Chile | -33.63790 | -78.85302 | KX817707 | KX817585 | KX817715 | KX817655 |
|  | *Philisca* | | *sp6* | E03 | MACN-Ar 28123 | Chile | -33.65276 | -78.84403 | KX817665 | KX817567 | KX817759 | KX817614 |
|  | *Philisca* | | *tripunctata* | E15 | CASENT 9016666 | Chile | -40.66639 | -72.17195 | KX817674 | KX817599 | KX817747 | KX817622 |
|  | *Philisca* | | *viernes* | E33/54 | MACN-Ar 28085 | Chile | -33.63790 | -78.85302 | KX817686 | KX817589 | KX817735 | KX817634 |
|  | *Sanogasta* | | *maculatipes* | sanma156/A023 | MACN-Ar-10588 | Argentina | -25.600889 | -65.632332 | **KX817502** | KR558869 | KR558816 | KR558932 |
|  | *Sanogasta* | | *xsignata* | sanxs07 | MACN-Ar-10776 | Argentina | -25.600889 | -65.632332 | KR559004 | KR558874 | KR558821 | KR558937 |
|  | *Selknamia* | | *minima* | A069 | MACN-Ar-29877 | Argentina | -54.85033 | -68.59602 | **KX817503** | **KX817420** | **KX817554** | **-** |
|  | *Tasata* | | *parcepunctata* | taspa021/A032 | MACN-Ar-10970 | Argentina | -34.225306 | -58.90025 | KR559006 | **KX817421** | KR558823 | KR558939 |
|  | *Tomopisthes* | | *horrendus* | tomho25 | MACN-Ar-10824 | Chile | -40.666278 | -72.173333 | KR559009 | KR558877 | KR558826 | KR558942 |
| Anyphaenidae, Anyphaeninae | | | | | | | | | | | | |
|  | | *Anyphaena* | *accentuata* | anyac039 | ARASP000039 | Belgium | 50.823881 | 4.518551 | KR559012 | KR558880 | KR558829 | KR558945 |
|  | | *Anyphaena* | *californica* | acalifornica1 | - | - | - | - | DQ628605 | - | DQ628633 | DQ628660 |
|  | | *Anyphaena* | *pacifica* | 08BARAC-0287 | - | - | - | - | KM834979 | - | KM225194 | KM225038 |
|  | | *Anyphaenoides* | *clavipes* | anycla019 | - | Argentina | -27.116667 | -54.8 | KR558955 | KR558838 | KR558772 | KR558889 |
|  | | *Aysha* | *proseni* | aypro08 | MACN-Ar-28890 | Uruguay | -34.317667 | -54.802972 | KR558963 | KR558843 | KR558780 | KR558897 |
|  | | *Buckupiella* | *imperatriz* | A139 | MACN-Ar-32443 | Argentina | -34.608401 | -58.35025 | **KX817504** | **KX817422** | **KX817555** | **KX817456** |
|  | | *Hatitia* | *sp* | ARAMR000428 | - | - | - | - | KX817505 | KX817423 | KX817556 | KX817457 |
|  | | *Hibana* | *sp* | MCH2003 | - | - | - | - | AY297422 | AY296713 | - | AY297295 |
|  | | *Jessica* | *osoriana* | jesos022 | - | Argentina | -27.116667 | -54.8 | KR558974 | KR558851 | KR558791 | KR558908 |
|  | | *Otoniela* | *adisi* | F01 | MACN-Ar-10981 | Argentina | -34.225306 | -58.90025 | KR558984 | KR558881 | - | KR558947 |
|  | | *Xiruana* | *gracilipes* | ARAMR000182 | MACN-Ar-28870 | Argentina | -27.116667 | -54.8 | KR559011 | KR558879 | KR558828 | KR558944 |
| Clubionidae | | | | | | | | | | | | |
|  | | *Elaver* | *sp* | ARAMR000163 | - | - | - | - | KX817506 | KX817424 | KX817557 | KX817458 |

**Table B.** Primers used for amplifying gene regions cytochrome oxidase c subunit I (COI), 16S rDNA (16S), 28S rDNA (28S), and Histone 3-a (H3a) sequences used in phylogenetic analyses of *Amaurobioides* and outgroup taxa.

| Marker | Primer name | Primer sequence (5’ to 3’) | Reference |
| --- | --- | --- | --- |
| COI | LCOI 1490 | GGTCAACAAATCATAAAGATATTGG | Folmer *et al.* (1994) |
|  | HCO extern B | CTATTGAWARAACATARTGAAAATG | Arango & Wheeler (2007) |
| 16S | 16S A | CGCCTGTTTATCAAAAACAT | Simon et al. (1994) |
|  | 16S B | CCGGTTTGAACTCAGATC | Simon et al. (1994) |
| H3a | H3a F | ATGGCTCGTACCAAGCAGACVGC | Colgan *et al.* (1998) |
|  | H3a R | ATATCCTTRGGCATRATRGTGAC | Colgan *et al.* (1998) |
| 28S | 28S O | GAAACTGCTCAAAGGTAAACGG | Hedin & Maddison (2001) |
|  | 28S C | GGTTCGATTAGTCTTTCGCC | Hedin & Maddison (2001) |

**Table C.** Partitioning scheme and nucleotide substitution models chosen for phylogenetic analyses of *Amaurobioides* and outgroup taxa.

| Subset Partitions | Best Model | Reference |
| --- | --- | --- |
| COI_1 | TrN+I+G | Tamura & Nei, 1993 |
| COI_2 | TVM+I+G | Posada, 2003 |
| COI_3 | HKY+G | Hasegawa *et al.*, 1985 |
| H3a_1, H3a_2 | TVMef+G | Posada, 2003 |
| H3a_3 | TVMef+G | Posada, 2003 |
| 16S | GTR+I+G | Lanave *et al.*, 1984 |
| 28S | GTR+I+G | Lanave *et al.*, 1984 |

**Table D**. Estimates of net evolutionary divergence between *Amaurobioides* species based on the sequences of the four markers used in this study for phylogenetic inferences

| Marker | *Amaurobioides* |  |  |  |  |  |  |  |  |
| --- | --- | --- | --- | --- | --- | --- | --- | --- | --- |
|  |  | *pleta* | *pallida* | spAfrica | *africana* | spFlinders | *chilensis* | *isolata* | *litoralis* |
| COI |  |  |  |  |  |  |  |  |  |
|  | *pleta* |  |  |  |  |  |  |  |  |
|  | *pallida* | 0.021 |  |  |  |  |  |  |  |
|  | spAfrica | 0.081 | 0.094 |  |  |  |  |  |  |
|  | *africana* | 0.094 | 0.110 | 0.057 |  |  |  |  |  |
|  | spFlinders | 0.071 | 0.087 | 0.103 | 0.104 |  |  |  |  |
|  | *chilensis* | 0.062 | 0.083 | 0.106 | 0.108 | 0.078 |  |  |  |
|  | *isolata* | 0.052 | 0.07 | 0.112 | 0.096 | 0.067 | 0.062 |  |  |
|  | *litoralis* | 0.064 | 0.084 | 0.103 | 0.110 | 0.034 | 0.076 | 0.067 |  |
|  | *maritima* | 0.047 | 0.053 | 0.106 | 0.100 | 0.073 | 0.055 | 0.049 | 0.070 |
| 16S |  |  |  |  |  |  |  |  |  |
|  | *pleta* |  |  |  |  |  |  |  |  |
|  | *pallida* | 0 |  |  |  |  |  |  |  |
|  | spAfrica | 0.078 | 0.078 |  |  |  |  |  |  |
|  | *africana* | 0.090 | 0.090 | 0.062 |  |  |  |  |  |
|  | spFlinders | 0.043 | 0.043 | 0.087 | 0.102 |  |  |  |  |
|  | *chilensis* | 0.027 | 0.026 | 0.069 | 0.078 | 0.009 |  |  |  |
|  | *isolata* | 0.034 | 0.034 | 0.087 | 0.093 | 0.037 | 0.043 |  |  |
|  | *litoralis* | 0.040 | 0.040 | 0.084 | 0.102 | 0.006 | 0.009 | 0.037 |  |
|  | *maritima* | 0.031 | 0.031 | 0.081 | 0.105 | 0.022 | 0 | 0.034 | 0.019 |
| H3a |  |  |  |  |  |  |  |  |  |
|  | *pleta* |  |  |  |  |  |  |  |  |
|  | *pallida* | - |  |  |  |  |  |  |  |
|  | spAfrica | 0.050 | - |  |  |  |  |  |  |
|  | *africana* | 0.044 | - | 0.005 |  |  |  |  |  |
|  | spFlinders | 0.055 | - | 0.011 | 0.016 |  |  |  |  |
|  | *chilensis* | 0.050 | - | 0.017 | 0.011 | 0.006 |  |  |  |
|  | *isolata* | - | - | - | - | - | - |  |  |
|  | *litoralis* | 0.055 | - | 0.011 | 0.016 | 0 | 0.006 | - |  |
|  | *maritima* | 0.012 | - | 0.012 | 0.006 | 0.007 | 0 | - | 0.007 |
| 28S |  |  |  |  |  |  |  |  |  |
|  | *pleta* |  |  |  |  |  |  |  |  |
|  | *pallida* | 0 |  |  |  |  |  |  |  |
|  | spAfrica | 0.038 | 0.038 |  |  |  |  |  |  |
|  | *africana* | 0.032 | 0.032 | 0.006 |  |  |  |  |  |
|  | spFlinders | 0.025 | 0.025 | 0.038 | 0.032 |  |  |  |  |
|  | *chilensis* | 0.025 | 0.025 | 0.038 | 0.032 | 0.019 |  |  |  |
|  | *isolata* | 0.019 | 0.019 | 0.032 | 0.025 | 0.019 | 0.019 |  |  |
|  | *litoralis* | 0.019 | 0.019 | 0.032 | 0.025 | 0.006 | 0.019 | 0.013 |  |
|  | *maritima* | 0.013 | 0.013 | 0.025 | 0.019 | 0.013 | 0.013 | 0.006 | 0.006 |

**Table E**. Mean rates (substitution/site/Myr) for molecular markers from this study’s concatenated Bayesian analyses compared to rates estimated in other studies of spiders

| COI | 16S | 28S | H3a | Study |
| --- | --- | --- | --- | --- |
| 0.01960 | 0.00355 | 0.00254 | 0.00355 | Present study |
| 0.0108 | 0.00476 | 0.00140 | 0.00182 | (Soto pers. obs.; BEAST) |
| 0.0173 | 0.0081 | 0.0019 | 0.0022 | (Soto pers. obs.; *BEAST) |
| 0.0168 | n/a | 0.0016 | 0.00066 | (Zhang & Li, 2013) |
| 0.0125 | 0.0063 | 0.0060 | 0.00108 | (Bidegaray-Batista & Arnedo, 2011) |
| 0.02649 | n/a | n/a | n/a | (Arnedo & Gillespie, 2006) |

**Table F.** Log-likelihoods (lnL), probabilities and significance values obtained from algorithms with different parameters (p= number of parameters; d = dispersal; e = extinction; j = founder effect) and dispersal scenarios (unconstrained = no dispersal constraints; distance = dispersal constraints based on distance; EWD = dispersal probabilities increased from east to west; WWD = dispersal probabilities increased from west to east) for estimates of the ancestral range and events of *Amaurobioides* based on the *BEAST species tree. D-stat denotes the significance between each method with and without the j parameter while 2lnBF are the Bayes Factors for the comparable methods between the four different scenarios (with 2 or 3 parameters: 2p and 3p, respectively)

| Scenario/method | p | d | e | j | lnL | D-stat | 2lnBF 2p | 2lnBF 3p |
| --- | --- | --- | --- | --- | --- | --- | --- | --- |
| Unconstrained | | | | | | | | |
| DEC | 2 | 0.0329 | 0.0945 | 0.0000 | -28.1558 |  | 7.8678 |  |
| DEC+J | 3 | 0.0001 | 0.0001 | 0.1194 | -17.1542 | 22.00** |  | 6.9190 |
| DIVALIKE | 2 | 0.0482 | 0.0592 | 0.0000 | -27.7031 |  | 6.9624 |  |
| DIVALIKE+J | 3 | 0.0001 | 0.0001 | 0.1234 | -17.4577 | 20.49** |  | 7.5260 |
| BAYAREALIKE | 2 | 0.0759 | 0.1830 | 0.0000 | -30.5489 |  | 12.6540 |  |
| BAYAREALIKE+J | 3 | 0.0001 | 0.0001 | 0.1109 | -17.8203 | 25.46** |  | 8.2512 |
| Distance | | | | | | | | |
| DEC | 2 | 0.0733 | 0.0927 | 0.0000 | -30.1210 |  | 11.7982 |  |
| DEC+J | 3 | 0.0001 | 0.0001 | 0.3459 | -19.1941 | 21.85** |  | 10.9988 |
| DIVALIKE | 2 | 0.0268 | 0.0498 | 0.0000 | -27.3049 |  | 6.1660 |  |
| DIVALIKE+J | 3 | 0.0001 | 0.0001 | 0.1257 | -17.2707 | 20.07** |  | 7.1520 |
| BAYAREALIKE | 2 | 0.0398 | 0.1606 | 0.0000 | -29.7908 |  | 11.1378 |  |
| BAYAREALIKE+J | 3 | 0.0001 | 0.0001 | 0.1109 | -17.8311 | 23.92** |  | 8.2728 |
| EWD | | | | | | | | |
| DEC | 2 | 0.0329 | 0.0945 | 0.0000 | -28.1558 |  | 7.8678 |  |
| DEC+J | 3 | 0.0001 | 0.0001 | 0.1194 | -17.1542 | 22.00** |  | 6.9190 |
| DIVALIKE | 2 | 0.1765 | 0.1009 | 0.0000 | -28.8012 |  | 9.1586 |  |
| DIVALIKE+J | 3 | 0.0127 | 0.0001 | 0.4130 | -18.4840 | 20.63** |  | 9.5786 |
| BAYAREALIKE | 2 | 0.1266 | 0.1819 | 0.0000 | -30.3500 |  | 12.2562 |  |
| BAYAREALIKE+J | 3 | 0.0148 | 0.0270 | 0.4145 | -23.5804 | 13.54** |  | 19.7714 |
| WWD | | | | | | | | |
| DEC | 2 | 0.0329 | 0.0945 | 0.0000 | -28.1558 |  | 7.8678 |  |
| DEC+J | 3 | 0.0001 | 0.0001 | 0.1194 | -17.1542 | 22.00** |  | 6.9190 |
| DIVALIKE | 2 | 0.0794 | 0.0001 | 0.0000 | -24.2219 |  | *** |  |
| DIVALIKE+J | 3 | 0.0001 | 0.0001 | 0.3653 | -13.6947 | 21.05** |  | *** |
| BAYAREALIKE | 2 | 0.1377 | 0.1675 | 0.0000 | -29.5996 |  | 10.7554 |  |
| BAYAREALIKE+J | 3 | 0.0001 | 0.0001 | 0.3090 | -13.6966 | 31.81** |  | 0.0038 |

** = p<0.001; *** highest log-likelihood for models with the same number of parameters

**Table G.** Log-likelihoods (lnL), probabilities and significance values obtained from algorithms with different parameters (p= number of parameters; d = dispersal; e = extinction; j = founder effect) and dispersal scenarios (unconstrained = no dispersal constraints; distance = dispersal constraints based on distance; EWD = dispersal probabilities increased from east to west; WWD = dispersal probabilities increased from west to east) for estimates of the ancestral range and events of *Amaurobioides* with four areas (Africa, Antarctica, Australasia and South America) based on the concatenated BEAST tree. D-stat denotes the significance between each method with and without the j parameter while 2lnBF are the Bayes Factors for the comparable methods between the four different scenarios (with 2 or 3 parameters: 2p and 3p, respectively)

| Scenario/method | p | d | e | j | lnL | D-stat | 2lnBF 2p | 2lnBF 3p |
| --- | --- | --- | --- | --- | --- | --- | --- | --- |
| Unconstrained | | | | | | | | |
| DEC | 2 | 0.0058 | 0.0001 | 0.0000 | -13.7929 |  | *** |  |
| DEC+J | 3 | 0.0001 | 0.0001 | 0.0696 | -9.7237 | 8.14* |  | 1.2574 |
| DIVALIKE | 2 | 0.0140 | 0.0001 | 0.0000 | -16.0781 |  | 4.5706 |  |
| DIVALIKE+J | 3 | 0.0001 | 0.0001 | 0.0764 | -9.8548 | 12.45** |  | 1.5196 |
| BAYAREALIKE | 2 | 0.0150 | 0.0763 | 0.0000 | -17.6160 |  | 7.6463 |  |
| BAYAREALIKE+J | 3 | 0.0001 | 0.0001 | 0.0719 | -10.2454 | 14.74** |  | 2.3008 |
| Distance | | | | | | | | |
| DEC | 2 | 0.0176 | 0.0001 | 0.0000 | -14.3076 |  | 1.0296 |  |
| DEC+J | 3 | 0.0001 | 0.0001 | 0.2839 | -10.5325 | 7.55* |  | 2.8749 |
| DIVALIKE | 2 | 0.0140 | 0.0001 | 0.0000 | -16.0781 |  | 4.5706 |  |
| DIVALIKE+J | 3 | 0.0001 | 0.0001 | 0.0764 | -9.8548 | 12.45** |  | 1.5196 |
| BAYAREALIKE | 2 | 0.0150 | 0.0763 | 0.0000 | -17.6160 |  | 7.6463 |  |
| BAYAREALIKE+J | 3 | 0.0001 | 0.0001 | 0.0719 | -10.2454 | 14.74** |  | 2.3008 |
| EWD | | | | | | | | |
| DEC | 2 | 0.0099 | 0.0001 | 0.0000 | -13.8810 |  | 0.1763 |  |
| DEC+J | 3 | 0.0001 | 0.0001 | 0.1811 | -10.9194 | 4.74 |  | 3.6487 |
| DIVALIKE | 2 | 0.0140 | 0.0001 | 0.0000 | -16.0781 |  | 4.5706 |  |
| DIVALIKE+J | 3 | 0.0001 | 0.0001 | 0.0764 | -9.8548 | 12.45** |  | 1.5196 |
| BAYAREALIKE | 2 | 0.0150 | 0.0763 | 0.0000 | -17.6160 |  | 7.6463 |  |
| BAYAREALIKE+J | 3 | 0.0001 | 0.0001 | 0.0719 | -10.2454 | 14.74** |  | 2.3008 |
| WWD | | | | | | | | |
| DEC | 2 | 0.0202 | 0.0182 | 0.0000 | -15.6853 |  | 3.7849 |  |
| DEC+J | 3 | 0.0001 | 0.0001 | 0.1275 | -9.0950 | 13.18** |  | *** |
| DIVALIKE | 2 | 0.0140 | 0.0001 | 0.0000 | -16.0781 |  | 4.5706 |  |
| DIVALIKE+J | 3 | 0.0001 | 0.0001 | 0.0764 | -9.8548 | 12.45** |  | 1.5196 |
| BAYAREALIKE | 2 | 0.0150 | 0.0763 | 0.0000 | -17.6160 |  | 7.6463 |  |
| BAYAREALIKE+J | 3 | 0.0001 | 0.0001 | 0.0719 | -10.2454 | 14.74** |  | 2.3008 |

* = p<0.01, ** = p<0.001; *** highest log-likelihood for models with the same number of parameters

**Matrix A.** Unconstrained dispersal multiplier matrix with dispersal probabilities between areas (AM = South America; AF = Africa; AU = South Australia; AT = Tasmania; NN = North Island and northern part of South Island of New Zealand; NS = central and southern part of South Island of New Zealand; AN = Antarctica).

**AM AF AU AT NN NS AN**

**AM 1 1 1 1 1 1 1**

**AF 1 1 1 1 1 1 1**

**AU 1 1 1 1 1 1 1**

**AT 1 1 1 1 1 1 1**

**NN 1 1 1 1 1 1 1**

**NS 1 1 1 1 1 1 1**

**AN 1 1 1 1 1 1 1**

**END**

**Matrix B.** Distance constrained dispersal multiplier matrix with dispersal probabilities between areas (AM = South America; AF = Africa; AU = South Australia; AT = Tasmania; NN = North Island and northern part of South Island of New Zealand; NS = central and southern part of South Island of New Zealand; AN = Antarctica).

**AM AF AU AT NN NS AN**

**AM 1 0.25 1e-05 0.05 0.2 0.2 0.9**

**AF 0.25 1 0.1 0.05 1e-05 1e-05 0.5**

**AU 1e-05 0.1 1 0.85 0.6 0.6 0.52**

**AT 0.05 1e-05 0.85 1 0.77 0.84 0.8**

**NN 0.2 1e-05 0.6 0.77 1 0.98 0.6**

**NS 0.2 1e-05 0.6 0.84 0.98 1 0.82**

**AN 0.9 0.5 0.52 0.8 0.6 0.82 1**

**END**

**Matrix C.** East-to-west (EWD) constrained dispersal multiplier matrix with dispersal probabilities between areas (AM = South America; AF = Africa; AU = South Australia; AT = Tasmania; NN = North Island and northern part of South Island of New Zealand; NS = central and southern part of South Island of New Zealand; AN = Antarctica).

**AM AF AU AT NN NS AN**

**AM 1 1e-05 1e-05 1e-05 1 1 0.001**

**AF 1 1 1e-05 1e-05 1e-05 1e-05 0.001**

**AU 1e-05 1 1 1e-05 1e-05 1e-05 0.001**

**AT 1e-05 1e-05 1 1 1e-05 1e-05 0.001**

**NN 1e-05 1e-05 1 1 1 1 0.001**

**NS 1e-05 1e-05 1 1 1 1 0.001**

**AN 0.001 0.001 0.001 0.001 0.001 0.001 1**

**END**

**Matrix D.** West-to-east (WWD) constrained dispersal multiplier matrix with dispersal probabilities between areas (AM = South America; AF = Africa; AU = South Australia; AT = Tasmania; NN = North Island and northern part of South Island of New Zealand; NS = central and southern part of South Island of New Zealand; AN = Antarctica).

**AM AF AU AT NN NS AN**

**AM 1 1 1e-05 1e-05 1e-05 1e-05 0.001**

**AF 1e-05 1 1 1e-05 1e-05 1e-05 0.001**

**AU 1e-05 1e-05 1 1 1 1 0.001**

**AT 1e-05 1e-05 1 1 1 1 0.001**

**NN 1 1e-05 1e-05 1e-05 1 1 0.001**

**NS 1 1e-05 1e-05 1e-05 1 1 0.001**

**AN 0.001 0.001 0.001 0.001 0.001 0.001 1**

**END**

**References**

Arango CP, Wheeler WC. Phylogeny of the sea spiders (Arthropoda, Pycnogonida) based on direct optimization of six loci and morphology. Cladistics 2007; 23: 255–293.

Arnedo MA, Gillespie RG. Species diversification patterns in the Polynesian jumping spider genus Havaika Prószyjski, 2001 (Araneae, Salticidae). Mol Phylogenet Evol. 2006; 41: 472–495.

Bidegaray-Batista L, Arnedo MA. Gone with the plate: the opening of the Western Mediterranean basin drove the diversification of ground-dweller spiders. BMC Evol Biol. 2011; 11: 317–331.

Colgan DJ, McLauchlan A, Wilson GDF, Livingston SP, Edgecombe GD, Macaranas J, Cassis G, Gray MR. Histone H3 and U2 snRNA DNA sequences and arthropod molecular evolution. Austr J Zool. 1998; 46: 419–437.

Folmer O, Black MB, Hoch W, Lutz RA, Vrijehock RC. DNA primers for amplification of mitochondrial Cytochrome c Oxidase subunit I from diverse metazoan invertebrates. Mol Mar Biol Biotechnol. 1994; 3: 294–299.

Hasegawa M, Kishino H, Yano T. Dating of the human-ape splitting by a molecular clock of mitochondrial DNA. J Mol Evol.1985; 22: 160–74.

Hedin MC, Maddison WP. A Combined Molecular Approach to Phylogeny of the Jumping Spider Subfamily Dendryphantinae (Araneae: Salticidae). Mol Phylogenet Evol. 2001; 18: 386–403.

Lanave C, Preparata G, Saccone C, Serio G. A new method for calculating evolutionary substitution rates. J Mol Evol. 1984; 20: 86–93.

Posada D. Using MODELTEST and PAUP* to select a model of nucleotide substitution. Curr Protoc Bioinformatics 2003. Chapter 6: Unit 6.5. doi: 10.1002/0471250953.bi0605s00.

Simon C, Frati F, Beckenbach A, Crespi B, Liu H, Flook P. Evolution, weighting, and phylogenetic utility of mitochondrial gene sequences and a compilation of conserved polymerase chain reaction primers. Ann Entomol Soc Am. 1994; 87: 651–701.

Tamura K, Nei M. Estimation of the number of nucleotide substitutions in the control region of mitochondrial DNA in humans and chimpanzees. Mol Biol Evol. 1993; 10: 512–26.

Zhang Y, Li S. Ancient lineage, young troglobites: recent colonization of caves by Nesticella spiders. BMC Evol Biol. 2013; 13: 183–192.

**
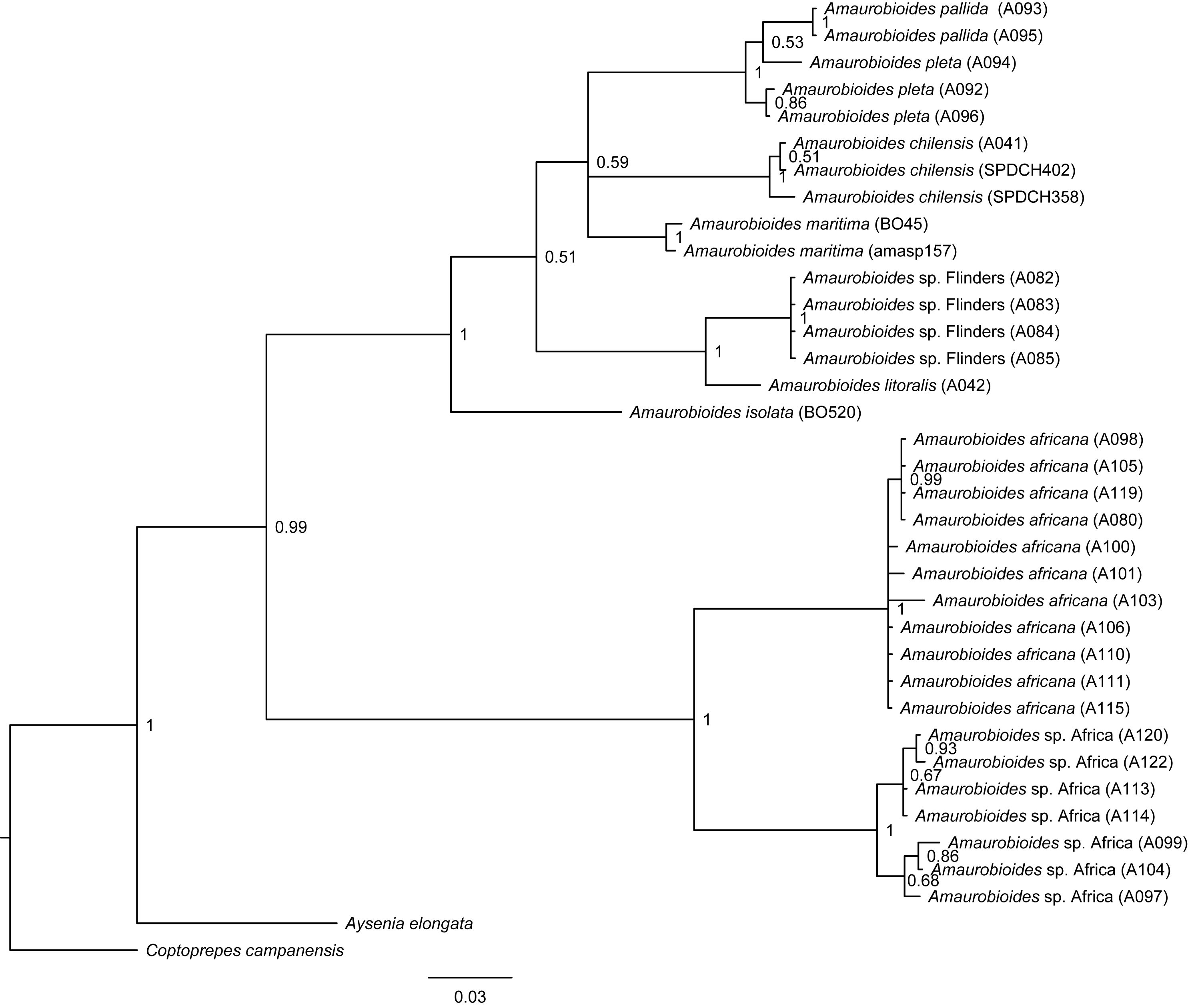
**

**Figure A.** Phylogenetic gene tree for *Amaurobioides* inferred by MrBayes for COI, obtained by 50% consensus of 10,000 trees. Bayesian posterior probabilities (PP) are shown at the nodes.

**
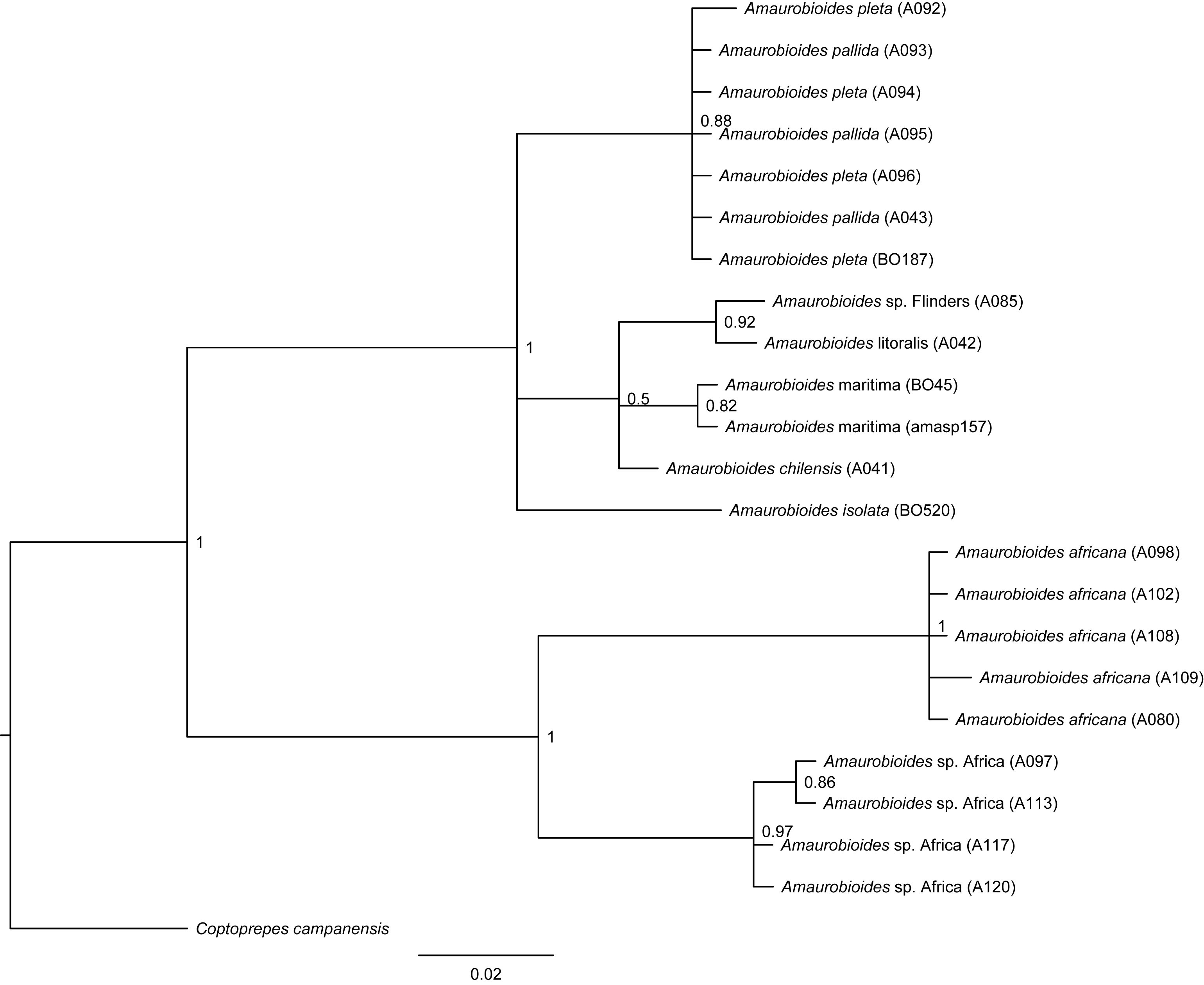
**

**Figure B.** Phylogenetic gene tree for *Amaurobioides* inferred by MrBayes for 16S, obtained by 50% consensus of 10,000 trees. Bayesian posterior probabilities (PP) >=0.9 are shown at the nodes.

**
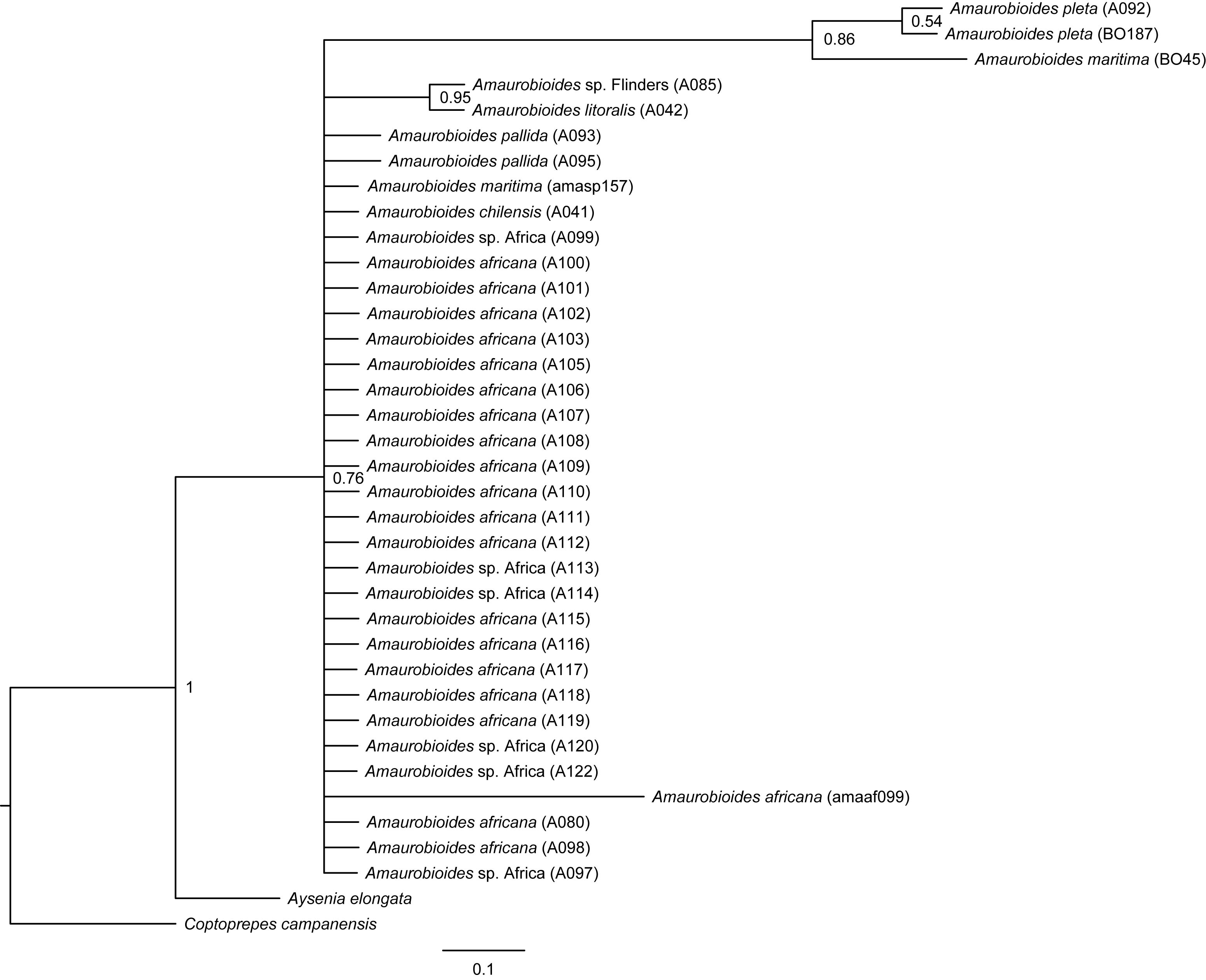
**

**Figure C.** Phylogenetic gene tree for *Amaurobioides* inferred by MrBayes for H3a, obtained by 50% consensus of 10,000 trees. Bayesian posterior probabilities (PP) >= 0.9 are shown at the nodes.

**
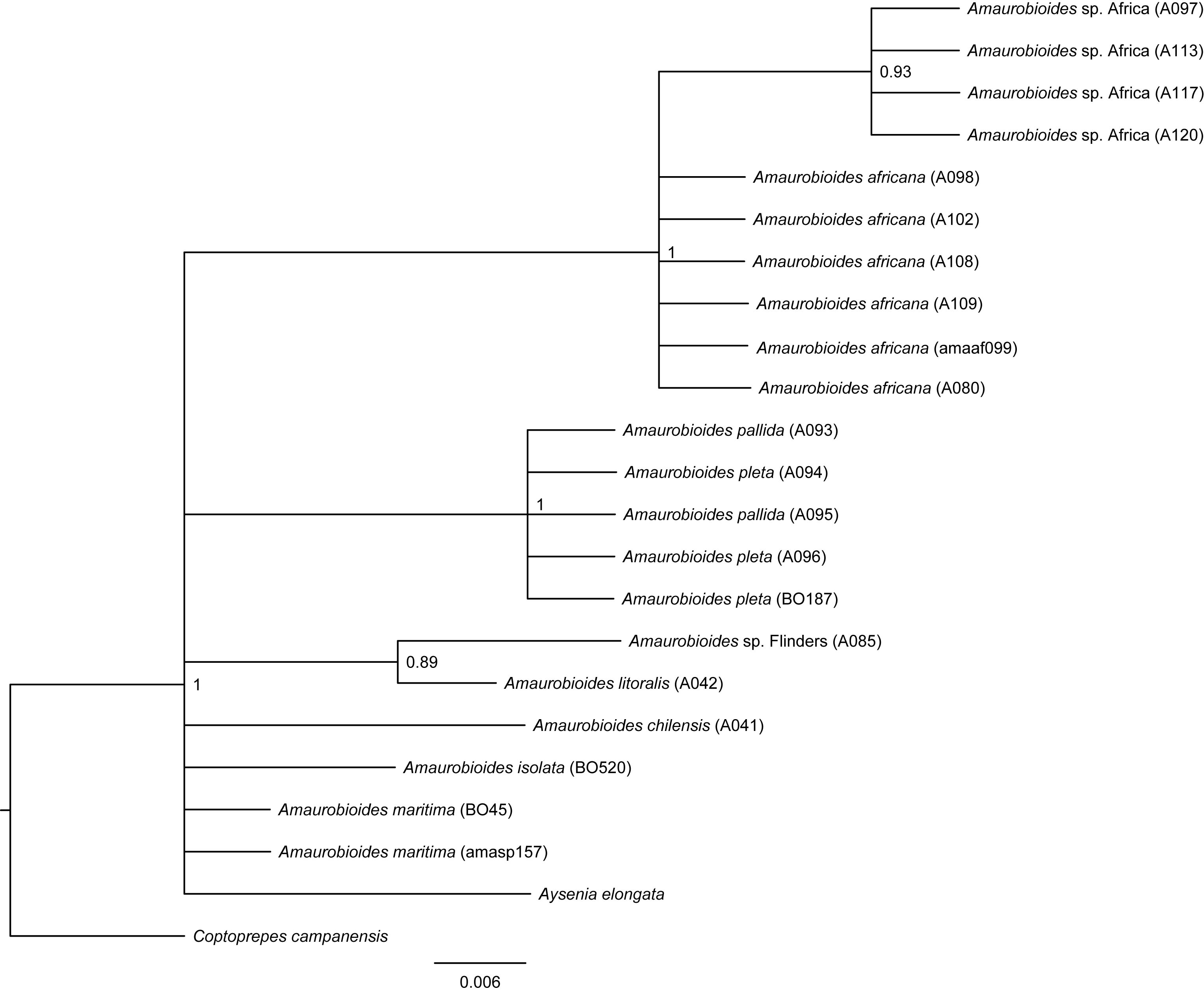
**

**Figure D.** Phylogenetic gene tree for *Amaurobioides* inferred by MrBayes for 28S, obtained by 50% consensus of 10,000 trees. Bayesian posterior probabilities (PP) >= 0.9 are shown at the nodes.


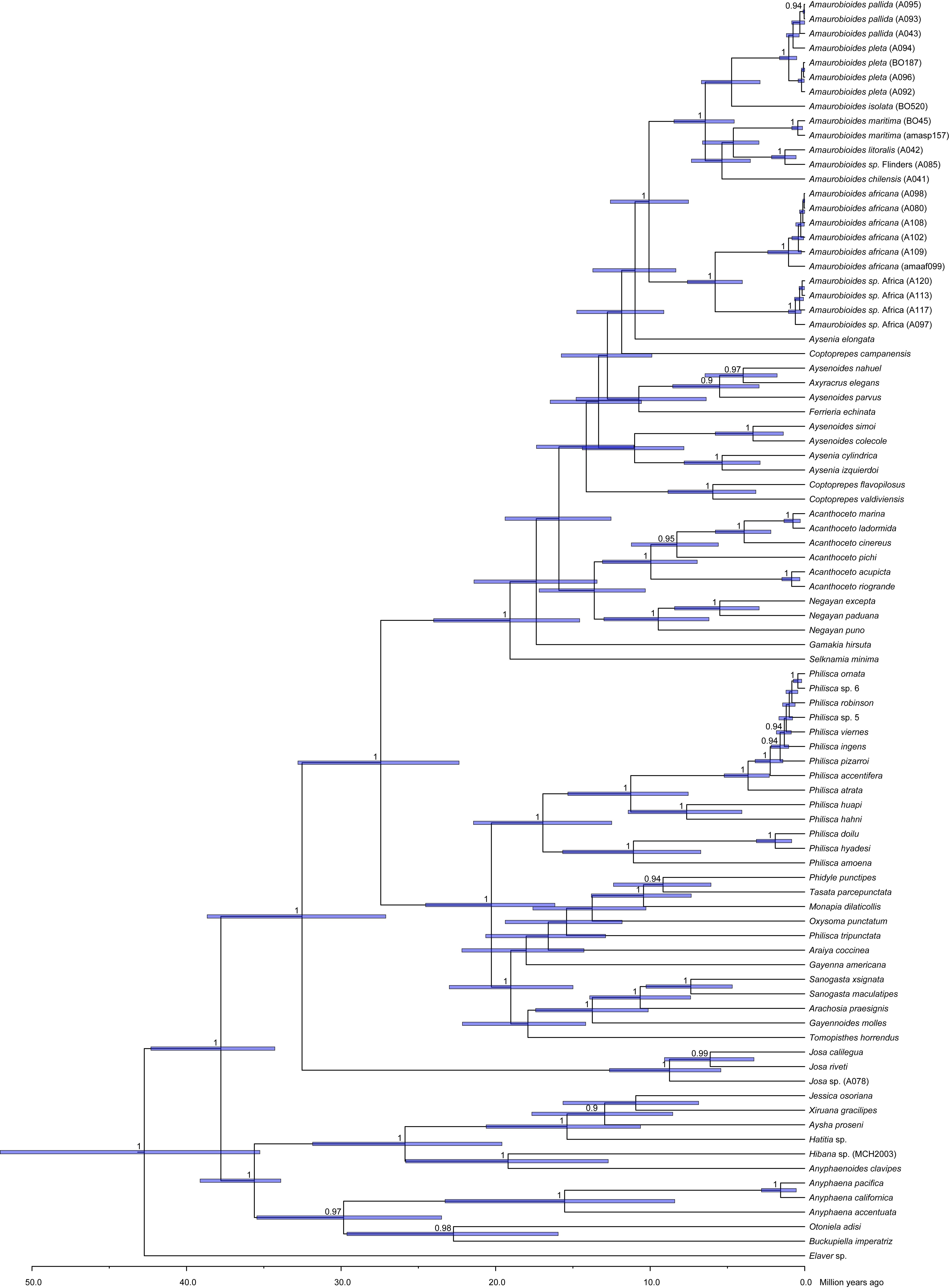


**Figure E.** Chronogram for *Amaurobioides* with node age estimates inferred by BEAST using the concatenated COI, 16S, H3a and 28S data obtained by selecting the maximum clade credibility tree from 10,000 trees. Bayesian posterior probabilities (PP) >= 0.9 are shown at the nodes, 95% Highest Posterior Density of node heights by blue bars. Timescale shown below the tree is in millions of years.


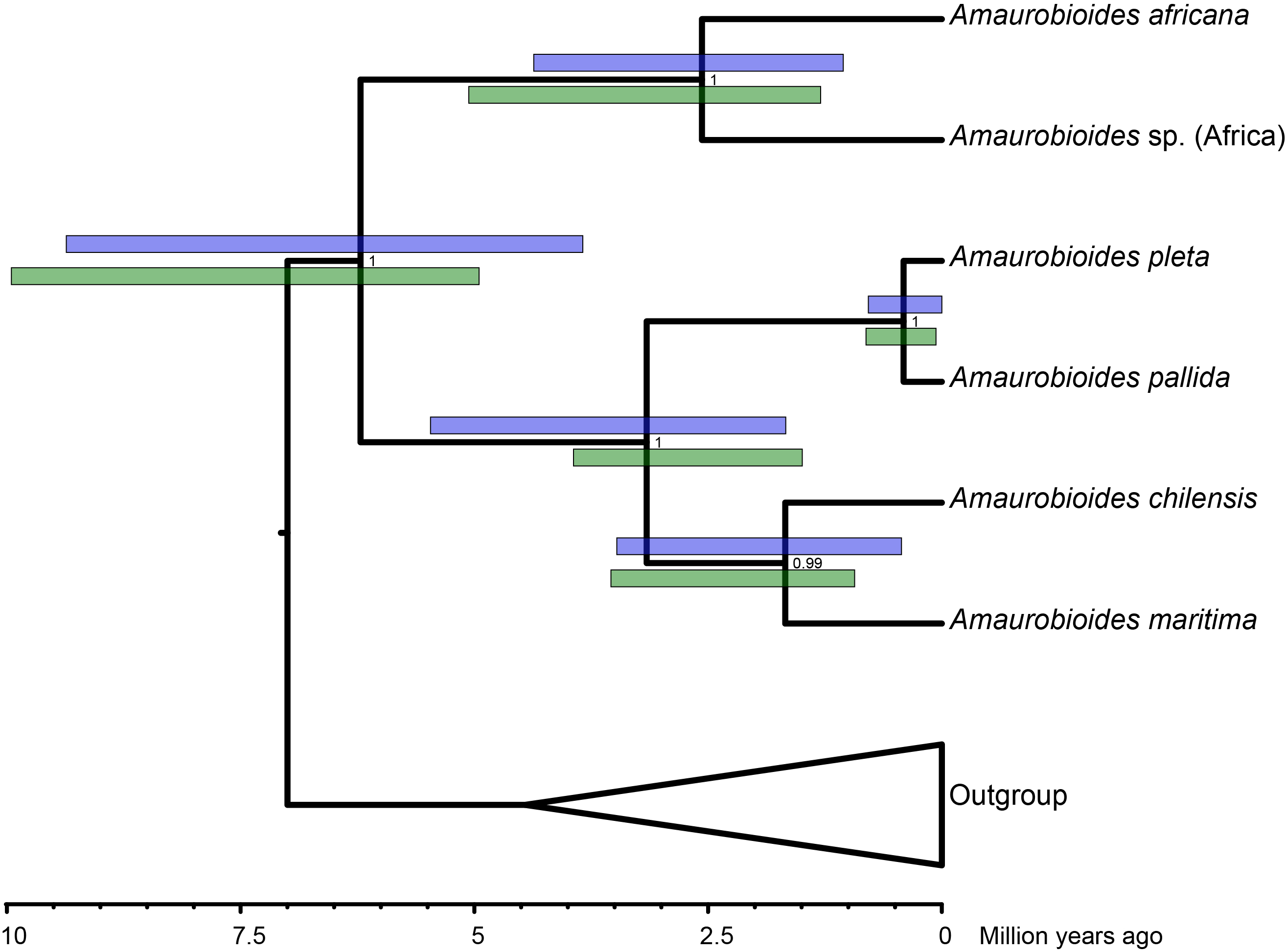


**Figure F.** Species coalescence tree with node age estimates based on the four markers COI, 16S, H3a and 28S, as inferred by *BEAST, reducing the number of terminal taxa to include only the *Amaurobioides* species with more than one representative. Blue bars at nodes represent the 95% Highest Posterior Density of the species tree analysis without single representatives of species and green bars the 95% Highest Posterior Density of the corresponding nodes from the full-taxon species tree (Fig 3).


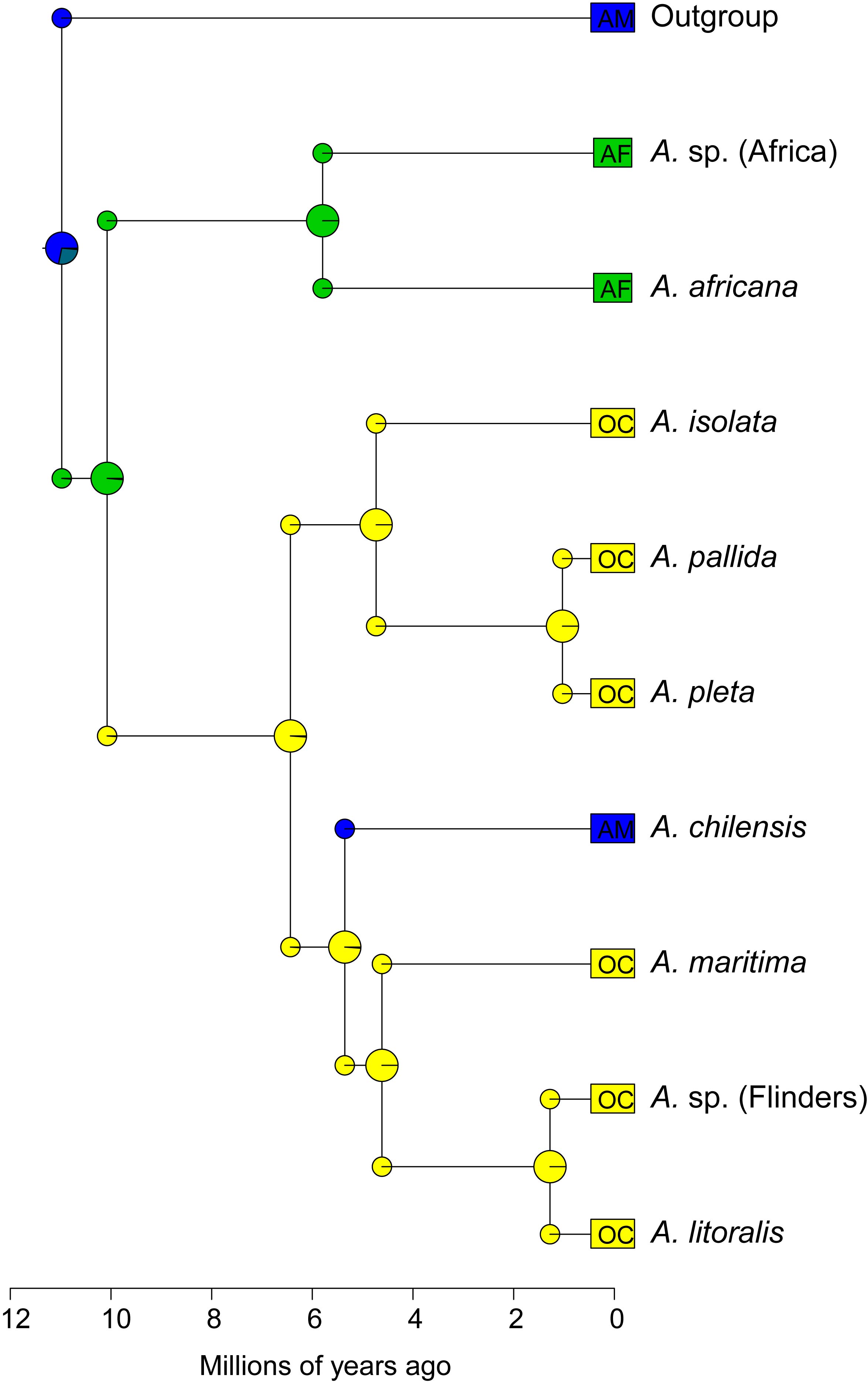


**Figure G.** Ancestral range estimates based on the DEC+J algorithm in BioGeoBEARS with the topology obtained from the concatenated analysis in BEAST for *Amaurobioides* species. Pies at nodes and edges show the relative probabilities of events and areas, colour-coded as in the boxes at the tips (AF = Africa, AM = South America, OC = Australasia; not shown: AN = Antarctica).
